# Gut redox and microbiome: charting the roadmap to T-cell regulation

**DOI:** 10.3389/fimmu.2024.1387903

**Published:** 2024-08-21

**Authors:** Sujata Prasad, Shilpi Singh, Samuel Menge, Iteeshree Mohapatra, Stefan Kim, Logan Helland, Gatikrushna Singh, Amar Singh

**Affiliations:** ^1^ Translational Division, MLM Labs, LLC, Oakdale, MN, United States; ^2^ Department of Neurosurgery, University of Minnesota, Minneapolis, MN, United States; ^3^ Department of Surgery, Schulze Diabetes Institute, University of Minnesota, Minneapolis, MN, United States; ^4^ Department of Veterinary and Biomedical Sciences, University of Minnesota, Saint Paul, MN, United States

**Keywords:** redox, T-cells, gut microbiome, reactive oxygen species, short-chain fatty acid, Treg cells, TRM cells, MAIT cells

## Abstract

The gastrointestinal (GI) tract redox environment, influenced by commensal microbiota and bacterial-derived metabolites, is crucial in shaping T-cell responses. Specifically, metabolites from gut microbiota (GM) exhibit robust anti-inflammatory effects, fostering the differentiation and regulation of CD8^+^ tissue-resident memory (TRM) cells, mucosal-associated invariant T (MAIT) cells, and stabilizing gut-resident Treg cells. Nitric oxide (NO), a pivotal redox mediator, emerges as a central regulator of T-cell functions and gut inflammation. NO impacts the composition of the gut microbiome, driving the differentiation of pro-inflammatory Th17 cells and exacerbating intestinal inflammation, and supports Treg expansion, showcasing its dual role in immune homeostasis. This review delves into the complex interplay between GI redox balance and GM metabolites, elucidating their profound impact on T-cell regulation. Additionally, it comprehensively emphasizes the critical role of GI redox, particularly reactive oxygen species (ROS) and NO, in shaping T-cell phenotype and functions. These insights offer valuable perspectives on disease mechanisms and potential therapeutic strategies for conditions associated with oxidative stress. Understanding the complex cross-talk between GI redox, GM metabolites, and T-cell responses provides valuable insights into potential therapeutic avenues for immune-mediated diseases, underscoring the significance of maintaining GI redox balance for optimal immune health.

## 1 Redox activity dynamics in the gastrointestinal tract: from mitochondria to microbes

The exploration of “Gut redox” focuses on the intricate redox equilibrium within the gastrointestinal (GI) tract, encompassing a dynamic interplay of various reactive species and antioxidants. This equilibrium plays a pivotal role in shaping gut health by influencing the composition of the gut microbiota, affecting inflammation, regulating nutrient absorption, ensuring mucosal protection, and contributing to the overall homeostasis of the gut immune system. A disturbance in the intricate equilibrium of gut redox can lead to oxidative stress, marked by an overabundance of reactive oxygen species (ROS) and reactive nitrogen species (RNS), potentially causing damage to cellular components. Although the gut is primarily anaerobic due to oxygen consumption by bacteria, certain gut cells like epithelial and immune cells can produce ROS. Immune cells, such as macrophages and neutrophils, activate NADPH oxidase (NOX) enzymes, generating superoxide radicals to combat pathogens. Epithelial cells can also produce ROS in response to inflammation, oxidative stress, or pathogen exposure. Additionally, ROS can result from dietary metabolism and interactions between gut microbes and host cells. Despite limited oxygen, ROS production in the gut plays crucial roles in both normal physiology and gastrointestinal pathologies.

The impact of ROS-associated signaling within GI tract is intricately linked to the localized concentration of ROS, leading to diverse outcomes. The various ROS, including hydrogen peroxide (H_2_O_2_), superoxide (O2^•-^), hydroxyl radical (OH^•^), and singlet oxygen, are generated through diverse intracellular pathways, with changes in oxygen partial pressure playing a significant role in creating distinct redox potentials throughout the GI tract (1, 2). Mitochondria is the primary source of ROS, generate superoxide as a byproduct of electron transport chain (ETC) activity. In this process, leaked electrons partially reduce oxygen (O_2_) to produce radicals such as O2^•-^ or H_2_O_2_. The accumulation of intracellular H_2_O_2_ induces the Fenton reaction, leading to the formation of highly reactive hydroxyl radicals (OH^•^) and posing a risk of cell and tissue damage (3–5). Excessive H_2_O_2_ can initiate uncontrollable chain reactions, triggering inflammatory responses (3, 6, 7).

**Figure 1:**
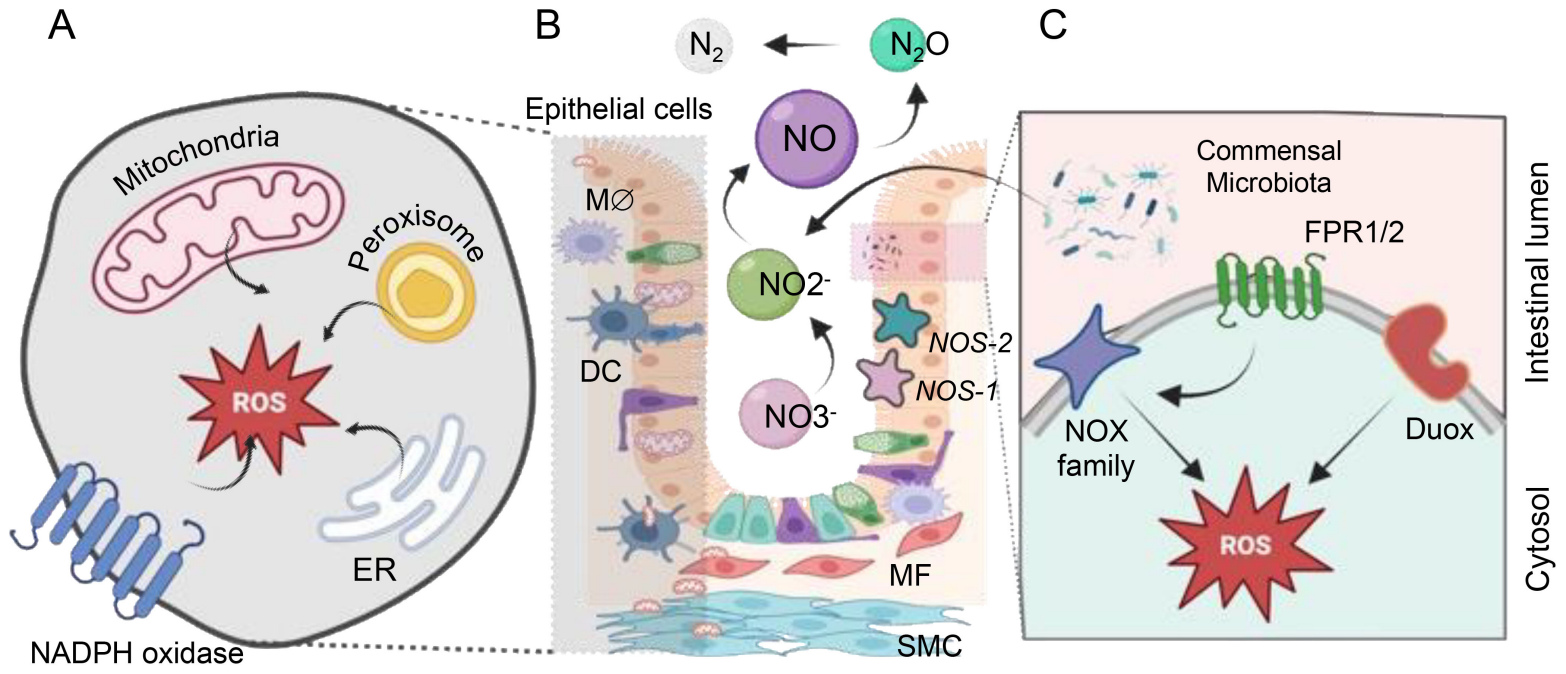
The sources of ROS and RNS within the gastrointestinal (GI) tract. ROS generation within the cell occurs in diverse compartments. **(A)** Mitochondria generate ROS through various mechanisms involving Complexes I to III (NADH in complex I or via FADH_2_ in complex II). Cytoplasmic ROS production predominantly relies on the NADPH oxidase (NOX) family proteins. The endoplasmic reticulum (ER) produces H_2_O_2_ by transferring electrons using a flavin adenine dinucleotide cofactor to oxygen. Additionally, peroxisomes generate H_2_O_2_ from OO_2_^•-^ through enzymes like ACOX and d-amino acid oxidase. **(B)** Commensal microbes significantly impact vital cellular processes by regulating the redox status within the gut. Nitric oxide (NO) is produced via nitrate reduction through bNOS activity found in *Bacillus* and *Streptomyces* spp. Additionally, gut bacteria like *E. coli* and *S. typhimurium* produce NO through enzymes such as nitrite reductase and nitrate reductase. Bacteria release formylated peptides recognized by formyl peptide receptors 1 and 2 (FPR1/2) on the apical surface of gut epithelial cells. These receptors activate NADPH oxidases, triggering localized ROS production to transduce microbial signals within the gut environment. **(C)** NADPH oxidase isoforms are localized in the GI mucosa, with NOX1 and DUOX2 predominantly found in the epithelium, NOX2 expressed in macrophages (Mϕ) and dendritic cells (DCs), and NOX4 present in myofibroblasts (MF), smooth muscle cells (SMC), and epithelium. Enzymatic NO generation from L-arginine by NO synthases (NOS-1, NOS-2) all of them expressed in gut endothelial and epithelial cells.

Apart from mitochondria, membrane-bound NOX are significant ROS generators, specifically crucial for immune cell functions (**Figure 1** **).** NOX1 and DUOX2, prevalent along the GI tract, produce substantial ROS quantities, essential for an oxidative burst employed by innate immune cells to eliminate phagocytized pathogens (8, 9). Additional enzymes in various cellular compartments, such as cyclooxygenases, lipoxygenases, and cytochromes P450 contribute to ROS generation (7, 10, 11). Notably, gut bacteria, including potent ROS generators like *lactobacilli*, are crucial in inducing controlled levels of ROS within human epithelial cells. *Lactobacillus* spp. likely regulate oxidative stress by influencing ROS-forming enzymes and modulating transcription factors like Nuclear factor erythroid-2-related factor 2 (NRF-2) and NF-κB (12). Studies in various models and clinical populations suggest that *Lactobacillus* could alleviate symptoms of conditions such as Inflammatory Bowel Disease (IBD), cancers, and liver damage by targeting inflammation and oxidative stress pathways (13). This highlights the potential therapeutic role of *Lactobacillus* in managing these health conditions.

Commensal gut bacteria can alter the intracellular redox environment, influencing essential cellular processes. Microbial signals activate NOX, inducing localized ROS production that influences redox sensor proteins in cells, thus affecting their activity (6, 12, 13).

Nitric oxide (NO) is a pivotal compound with intricate roles in the functioning of the intestinal microflora and beyond. It contributes to complex signaling processes in both eukaryotic cells and among gut microorganisms (**Figure 1**). The mammalian cells’ ability to produce NO through the enzyme Nitric oxide synthase (NOS) underscores its significance as a critical signaling molecule in the gastrointestinal tract (14). However, the potential accumulation of NO can lead to the formation of highly damaging peroxynitrite (ONOO^-^), which, in turn, induces cellular damage (15). Beyond eukaryotic cells, various commensal bacteria contribute to NO production through different pathways, such as bacterial NOS (bNOS) activity and dissimilatory nitrate reduction to ammonium (DNRA) (16). The generation of RNS and ROS by pathogenic bacteria contrasts with the actions of commensal gut bacteria, which play a role in reducing harmful RNS, rendering them less reactive. Unlike pathogenic bacteria that produce RNS and ROS, commensal bacteria such as *Escherichia coli, Lactobacilli, and Bifidobacteria* are involved in scavenging these reactive species rather than producing them. For instance, these commensal bacteria can execute denitrification, converting nitrite (NO_2_^-^) to NO and further to nitrous oxide (N_2_O). Enzymes like periplasmic and cytoplasmic nitrite reductases contribute to NO production through the reduction of nitrite (17). Lactic acid-producing bacteria generate NO through non-enzymatic reduction, while *E. coli* and *Salmonella typhimurium* employ biological pathways, utilizing nitrite reductase and nitrate reductase enzymes, respectively (16, 18). These mechanisms highlight the diverse strategies employed by gut commensal bacteria to mitigate the harmful effects of RNS and ROS in the GI tract.

## 2 Gastrointestinal redox equilibrium: the interplay with gut microbiota (GM) and metabolites

The redox balance within the GI tract, mirroring other physiological systems, is a delicate equilibrium between oxidation and reduction reactions. This balance is regulated by antioxidants and antioxidative enzymes, maintaining a controlled redox equilibrium under normal physiological conditions (5). This essential equilibrium allows for controlled oxidative signaling while preventing oxidative stress and the consequential damage to macromolecules such as proteins, lipids, and DNA. The transition from redox balance to oxidative stress depends on the context, exhibiting variations among tissues and cells due to their unique cellular backgrounds and antioxidant levels (19, 20). These antioxidants (enzymatic and non-enzymatic) play critical roles in the defense system. Glutathione (GSH) and thioredoxins are potent non-enzymatic antioxidants, scavenging reactive compounds and regulating redox-sensitive transcription factors (21, 22). Enzymatic defense involves key players like superoxide dismutases (SOD1/2/3), catalase (CAT), glutathione peroxidase (GPx), and glutathione reductase (GR) (23). CAT and SODs convert reactive species like O2^•-^ and H_2_O_2_ to less harmful substances (21). On the other hand, GPx plays a vital role in the glutathione-REDOX system by converting GSH to its oxidized form GSSG, effectively reducing harmful substances such as H_2_O_2_ and lipid hydroperoxides. The coordinated action with glutathione disulphide reductase (GSR) ensures the recycling of oxidized glutathione, highlighting the significance of these enzymes in maintaining cellular redox balance and combating oxidative stress (21). **Table 1** summarizes some common ROS, RNS, and cellular antioxidants.

**Table 1:** Types of reactive species and cellular antioxidants.

Reactive species	Radical	Non-Radical	References
Reactive oxygen species (ROS)	Superoxide (O_2_^•−^) Singlet oxide (O_2_^•−^) Alkoxyl (RO.) Hydroxyl radicals (^•^OH) Hydroperoxyl radical (HO_2_) Peroxyl (ROO.)	Hydrogen peroxide (H_2_O_2_) Hypocholorus acid (HOCl) Ozone (O_3_) Singlet oxygen (O_2_) Oxygen bi-radical (O_2_.) Aldehydes (HCOR)	(23–27)
Reactive nitrogen species (RNS)	Nitric oxide (NO^•^) Nitrogen dioxide (NO_2_.)	Peroxynitrite (ONOO^−^) Alkyl peroxynitrite (ROONO) Dinitrogen trioxide (N_2_O_3_) Dinitrogen tetroxide (N_2_O_4_) Nitrous acid (HNO_2_) Nitronium ion (NO_2_) Nitroxyl anion (NO.) Nitrosyl cation (NO^+^) Nitryl chloride (NO_2_Cl)	(28–31)
Antioxidants	Type of antioxidants	Target reactive species	References
Non-enzymatic	Glutathione (GSH)	Hydrogen peroxide	(32–36)
	Vitamin C	Superoxide, hydroxyl, reactive nitrogen species	
	Vitamin E	Lipid peroxides	
	Bilirubin	Lipid peroxides	
Enzymatic	Superoxide dismutase (SOD)	Superoxide	
	Catalase (CAT)	Hydrogen peroxide	
	Glutathione peroxidase (GPx)	Hydrogen peroxide, lipid peroxides	
	Peroxiredoxins	Hydrogen peroxide	
	Heme oxygenase	Heme	
	Ferritin	Free iron	

The antioxidant system actively maintains the redox balance, safeguarding the GI tract from oxidative stress-induced damage (21). Maintaining a balance in the GI tract is essential for proper functioning and overall health. Alterations in both the gut GM and gut metabolites can impact the GI redox balance in diverse ways. The diverse bacterial community in the gut exerts a multifaceted role, contributing to antioxidant defense and the preservation of redox balance (**Table 2**). Notably, specific bacterial strains produce antioxidant enzymes, and their metabolic activities yield crucial elements like hydrogen gas (H_2_) and short-chain fatty acids (SCFAs). These substances play pivotal roles in mitigating oxidative stress, fortifying the gut epithelial barrier, and modulating immune responses (55–57). The gut microbiota’s profound impact on immune system development and function, along with its ability to metabolize xenobiotics and produce mucin-degrading enzymes, underscores its role in reducing oxidative stress and maintaining a protective mucus layer. Recognizing the complexity and variability of the gut microbiota among individuals is crucial, with dysbiosis potentially contributing to disruptions in redox balance, oxidative stress, and GI diseases.

**Table 2:** Gut microbiota-induced ROS formation, major source, and associated effects.

Gut microbiota	Gut microbiota-induced ROS formation	ROS types	Enzymes involved in ROS generation	Organelles associated with ROS	ROS-mediated effects	Antioxidants of the host system involved in ROS regulation	References
*Lactobacillus* *Bifidobacterium* strains	Induced by bacterial metabolites and antigens	H_2_O_2_	Myeloperoxidase/Flavoproteins	Lysosomes/Peroxisomes	Trigger Neutrophile Promote pathogen defense Neutralize bacterial pathogens	Catalase Glutathione peroxidase Glutathione reductase	(5, 20, 37–41)
*E. coli*		O_2_·^−^	NADPH oxidases Mitochondria Complex I & III	Membrane (Plasma and vesicular)/ Mitochondria	Stimulate cellular signaling, inflammation, oxidative burst and host defense	Superoxide dismutase	(5, 42–44)
*E. coli* *Lactobacillus* *Bifidobacterium*	Degradation of purine, bacterial metabolite, and antigens	O_2_·^−^/ H_2_O_2_	Xanthine oxidase	Peroxisomes/Cytosol	Stimulate inflammation Neutralize bacterial pathogens,	Catalase Glutathione peroxidase Glutathione reductase Superoxide dismutase	(45–50)
*Lactobacilli bifidobacteria* *Escherichia coli, Bacteroides thetaiotaomicron* *Clostridium difficile*	Bacteria-derived lipopolysaccharide	NO	Inducible-nitric oxide synthase	Cytosol	Activate cellular signaling	Glutathione N-acetyl-L cysteine	(51–54)

Essentially, cultivating a well-balanced gut microbiota is crucial for gastrointestinal health, and this can be achieved through thoughtful dietary choices, lifestyle adjustments, and interventions like prebiotics and probiotics. Recognizing the significance of a healthy and diverse gut microbiota in sustaining a harmonious gut redox environment is fundamental for overall well-being. Through these strategies, we can support a resilient gut microbiota, reduce unnecessary inflammation, and protect against disruptions in redox balance, ultimately promoting optimal health.

## 3 GI redox dynamics: a key player in immune system modulation

**Figure 2:**
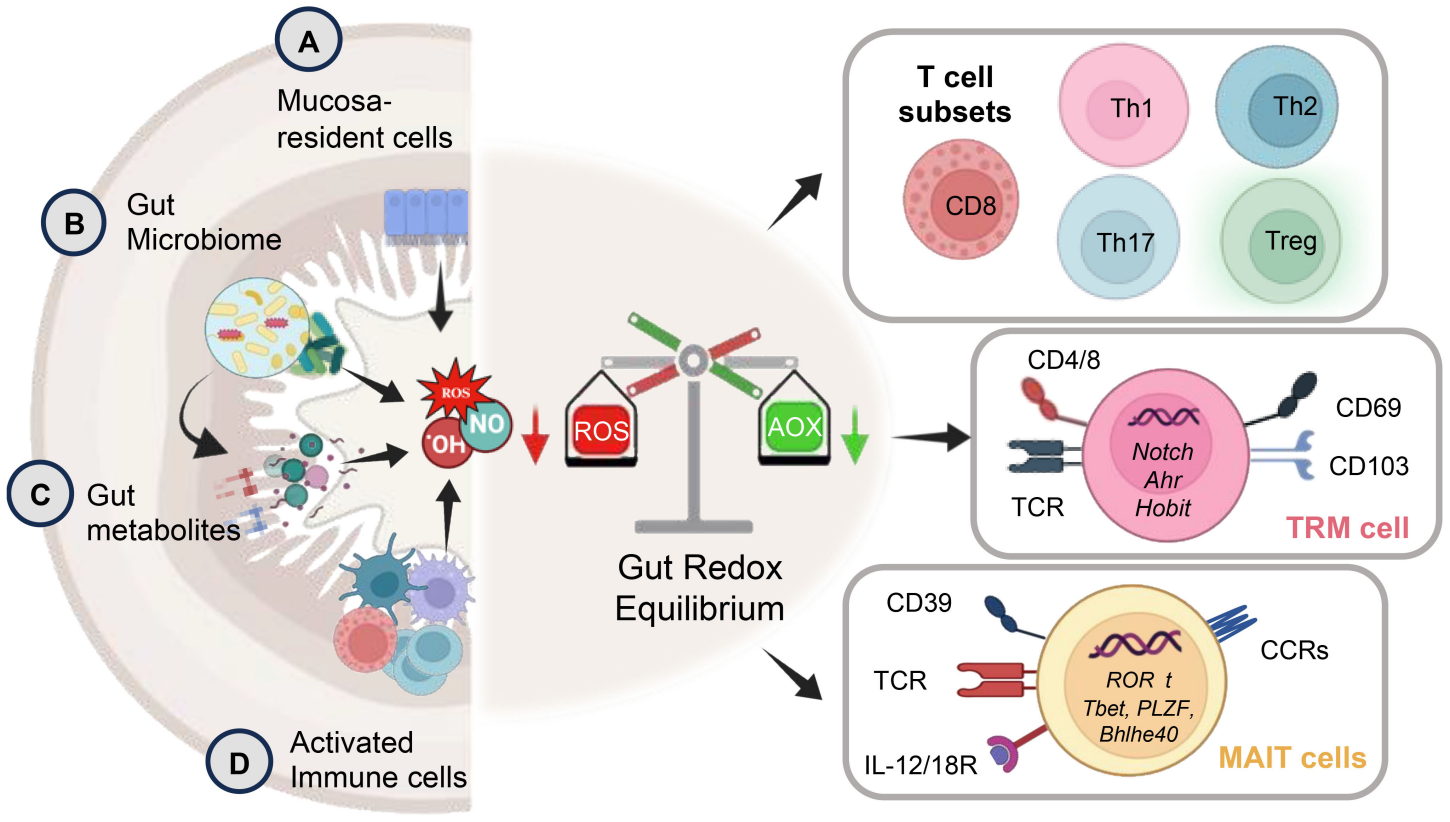
The schematic depicts key aspects of gut redox equilibrium and T-cell regulation gastrointestinal (GI) tract. The gut’s redox equilibrium and T-cell regulation are influenced by various cellular sources of reactive oxygen species (ROS) and reactive nitrogen species (RNS). The release ROS, RNS, or H_2_S, whether externally by **(A)** mucosa-resident cells, **(B)** gut bacteria, **(C)** pro and anti-inflammatory metabolites generated post-food digestion and bacterial fermentation, or **(D)** newly recruited innate immune cells (activated granulocytes and macrophages) during inflammation, or internally by chronically stimulated T-cells plays a crucial role. The gut redox is pivotal in maintaining a balance between T-cell subsets, modulating their activation and inactivation, thereby governing gut homeostasis and inflammation. The right panel shows key subsets of T-cell including tissue resident memory T-cells (TRM) and mucosal associated invariant T-cells (MAIT) with their key phenotypic identification marker and transcription factors. These T-cell subsets are profoundly influenced by gut redox dynamics.

Recent discoveries underscore the pivotal regulatory role of cellular redox status within the GI tract in shaping various aspects of immune function. This intricate influence extends to immune cell activation or inhibition, with outcomes often dictated by the concentration of redox factors. Oxidative stress, a consequence of imbalances in redox status, significantly impacts macromolecules, contributing to a spectrum of ailments such as inflammatory diseases, autoimmune disorder, aging, cancer, and neurodegeneration. The unique contribution of ROS as a third signaling entity, akin to proinflammatory cytokines, is a noteworthy discovery (58). Functioning alongside these cytokines, ROS acts as a crucial third signal that enhances T-cell proliferation, thereby influencing immune outcomes. This third signaling dimension, as observed in studies (59, 60), underscores the intricate orchestration required for a robust immune response. The release of ROS and RNS, whether originating from exogenous sources such as gut bacteria or mucosa-resident cells, or endogenously from chronic stimulated T-cells, plays a critical role in maintaining a delicate balance between T-cell activation and inactivation. This balance is essential for the precise regulation of immune responses, highlighting the nuanced control required for optimal immune function (9, 61) (**Figure 2**).

The delicate equilibrium of redox status emerges as a critical determinant in orchestrating the complex interplay between the GI epithelial barrier and immune cells. This finely tuned redox balance significantly influences macrophage (M1 and M2) polarization, shaping the ensuing immune response. Elevated oxidative stress tends to promote a pro-inflammatory M1 phenotype, while a more reduced environment facilitates M2 polarization, fostering tissue repair and immune regulation (62). The profound impact of redox balance extends to dendritic cells (DC), neutrophils, natural killer (NK/iNKT) cells, and innate lymphoid cells (ILCs), all pivotal players in the intricate web of mucosal immunity. Redox signaling modulates their functions, impacting processes such as antigen presentation, microbial killing, cytotoxic activity, and maintenance of gut homeostasis. Moreover, the influence of redox balance reaches into the realm of adaptive immunity, shaping the characteristics and functionality of B and T-cells (63). The redox state of the GI environment intricately modulates the equilibrium between pro-inflammatory Th1 and anti-inflammatory Th2 responses. While oxidative stress favors Th1 responses, a more reduced state may promote Th2 responses (63). Additionally, gut redox profoundly influences the generation and function of regulatory T-cells (Tregs), crucial for maintaining immune tolerance (64) (**Figure 2**).

The unique composition of the gut microbiome, individualized for each person, plays a pivotal role in maintaining a beneficial redox balance that suppresses inflammation. Continuous immune system activation in the intestine through direct contact with the microbiota underscores the intricate balance of pro- and anti-inflammatory mechanisms influenced by these microbial communities (5, 65). Gut commensal bacteria play a role in creating an antioxidative environment, suppressing inflammation, whereas pathogenic microbiota promote inflammation and alter the redox balance toward a pro-oxidative state (66). The anaerobic conditions in the gut are essential for maintaining homeostasis by creating an environment that supports the growth of beneficial anaerobic bacteria while inhibiting the proliferation of harmful aerobic bacteria. Anaerobic bacteria are well-adapted to the low-oxygen environment of the gut and play a vital role in various physiological processes, such as digestion, nutrient absorption, and immune regulation. By fermenting dietary fibers and other complex carbohydrates, anaerobic bacteria produce SCFAs and other metabolites that help nourish the gut epithelium, modulate T-cell responses, and maintain barrier integrity. Additionally, anaerobic bacteria contribute to gut homeostasis by consuming oxygen, which helps maintain the anaerobic conditions necessary for their survival. In contrast, dysbiosis, characterized by an imbalance in the gut microbiota composition, can disrupt this anaerobic environment. Factors such as inflammation, antibiotic use, and dietary changes can lead to dysbiosis, resulting in an overgrowth of aerobic bacteria and a decrease in beneficial anaerobic bacteria. Anaerobic probiotics, such as certain strains of *Lactobacillus*, *Bifidobacterium*, and *Faecalibacterium prausnitzii* have been shown to help restore gut homeostasis by replenishing beneficial bacteria and promoting an anaerobic environment (67, 68). These probiotics can scavenge oxygen and produce metabolites that support the growth of anaerobic bacteria while inhibiting the proliferation of harmful aerobic bacteria. By restoring microbial balance and enhancing gut barrier function, anaerobic probiotics can help prevent inflammation and maintain gut health. The ability of these species to augment and extend antigen-specific proliferative responses in T-cells emphasizes their crucial role in fine-tuning immune outcomes. Moreover, the intricate balance of redox status significantly influences the integrity of the epithelial barrier and its interactions with immune cells. The delicate interplay between redox balance and the adaptive immune system further extends to shaping T-cell characteristics, metabolic responses, and functionality, thus impacting the equilibrium between pro-inflammatory and anti-inflammatory responses.

## 4 Redox dynamics: impact on T-cell modulation

The influence of ROS on T-cells is intricate, demonstrating a dual role with both positive and negative impacts, contingent on the concentration of ROS and the specific context. Controlled ROS production is pivotal for T-cell activation and immune responses, while excessive or prolonged oxidative stress can contribute to T-cell dysfunction, thereby contributing to immune-related disorders. The maintenance of Redox equilibrium emerges as a critical factor for optimal immune system functionality, with oxidative signals playing a decisive role in initiating or concluding immune responses. The interplay between the gut microbiota and ROS holds significant importance in shaping the immune system. A balanced microbiome plays a key role in preserving a stable Redox balance, while dysbiosis disrupts this equilibrium, directly influencing various facets of T-cell function. The gut microenvironment, impacted by oxidative stress, influences T-cell recruitment, activation, and effector functions. Moreover, ROS regulation contributes to the delicate balance of Th1/Th2 responses, with elevated ROS levels influencing cell counts and gene expression patterns (60).

### 4.1 Redox control at the crossroads: antigen presentation and T-cell activation

The intricate interplay between redox signaling and immune modulation is evident in the modulation of T-cell receptor (TCR) and interleukin-2 (IL-2) receptor signaling pathways by ROS. Antigen-presenting cells (APCs), including macrophages and DCs, generate ROS during phagocytosis and antigen processing, influencing the activation and differentiation of T-cells upon antigen presentation (69, 70). Costimulatory molecules on APC surfaces, such as CD80 and CD86, are modulated by ROS, impacting the adaptive immune cell activation (71). The role of NOX2-derived ROS in DCs and their influence on CD8^+^ T-cell activation highlights the complex interplay within the immune response. In the context of viral infections, toll like receptor (TLR) signaling-induced ROS production in monocytes contributes to the upregulation of inhibitory costimulatory molecules, affecting immune escape. Notably, antioxidants like N-acetyl cysteine (NAC) and polyphenols counteract these effects, emphasizing the potential therapeutic implications (72). The generation of ROS in macrophages through FcγR I/FcγR III adds an additional dimension to immune regulation, impacting humoral immune responses during liver graft rejection (73, 74).

**Figure 3:**
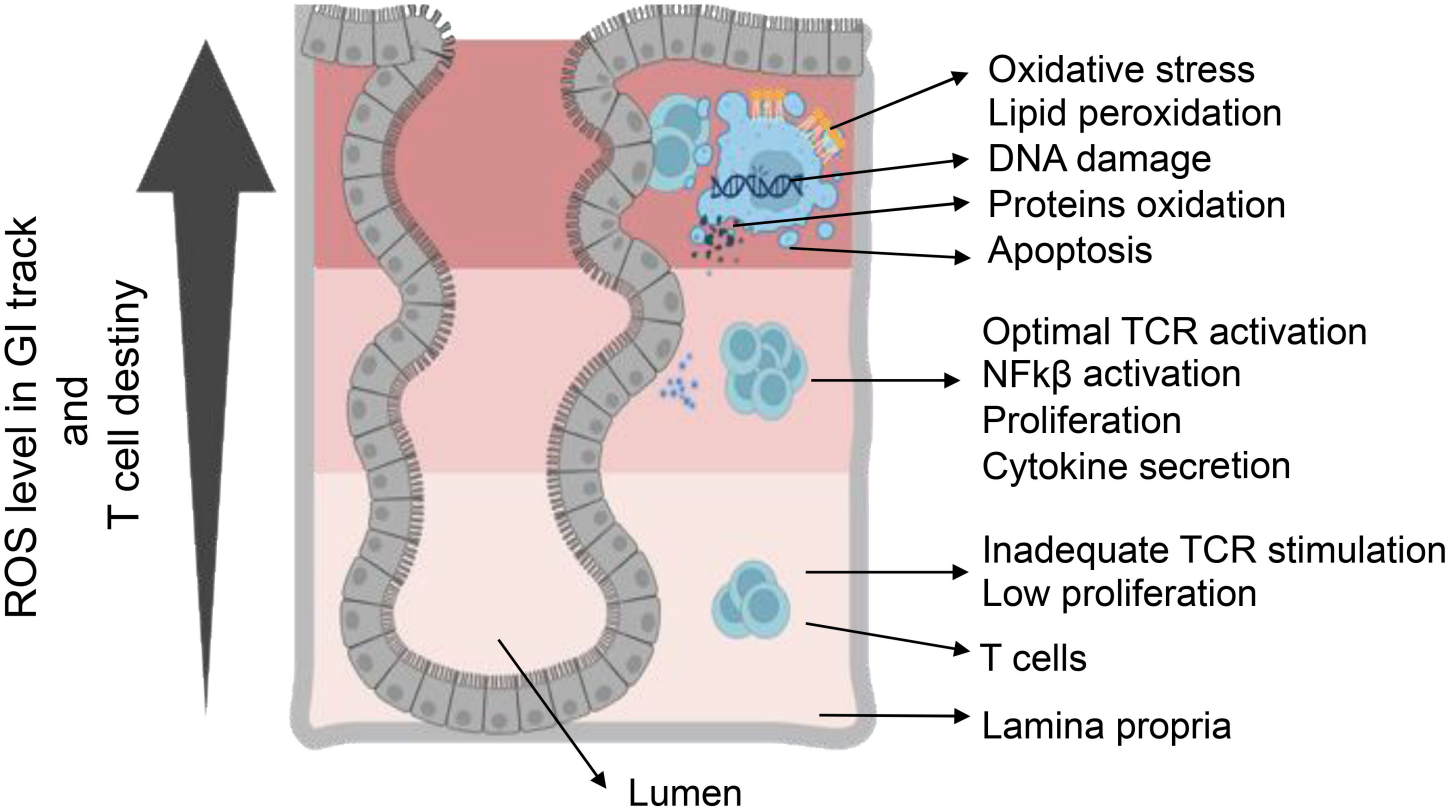
T-cell destiny in reaction to varied levels of ROS. Elevating ROS concentration triggers a varied T-cell response, involving TCR activation and cytokine production. Conversely, low ROS concentration leads to inadequate signaling, resulting in reduced activation and proliferation. Achieving optimal ROS conditions is essential for the appropriate activation of T-cells. ROS-induced oxidative stress causes cellular damage through DNA damage, protein oxidation, and lipid peroxidation. Furthermore, ROS participate in regulating the pro-inflammatory response mediated by HIF-α, NF-κB, and Nrf2. Serving as crucial signaling molecules, ROS bridges innate and adaptive immunity, influencing cellular behaviors such as metabolism, differentiation, and apoptosis.

The role of ROS in phagosomes is pivotal for pathogen elimination and optimal antigen presentation. NOX2-derived ROS play a crucial role in modulating the redox microenvironment and influencing antigenic peptide stability, ultimately enhancing T-cell activation (69). Conversely, reduced ROS levels in macrophages and DCs compromise CD8^+^ T-cell activation due to impaired antigen processing (75, 76). Furthermore, ROS can directly impact antigen structure, potentially contributing to autoimmune diseases (77). Mitochondrial ROS (mtROS) exhibit complex effects on antigen presentation (69). While increased mtROS in aged DCs hinders cross-presentation, mtROS-driven pH alkalization in plasmacytoid DCs (pDCs) is crucial for cross-presentation, demonstrating context-dependent influences (78). Within pDCs, ROS also participates in reacting to damage-associated molecular pattern molecules, influencing their stimulation capacity. The synergy between NOX2-derived ROS and mtROS, observed in macrophages, adds another layer of complexity to the modulation of immune responses (79, 80). Low levels of ROS serve as signaling molecules during T-cell activation, contributing to controlled immune responses. However, high levels of ROS can lead to T-cell dysfunction and activation-induced cell death, impacting the delicate balance between immune activation and tolerance (81). Chronic exposure to ROS may induce T-cells hypo-responsiveness, highlighting the potential implications for immune-related disorders (1, 2) (**Figure 3**). Overall, the intricate relationship between redox signaling and T-cell modulation underscores its multifaceted role in immune regulation.

### 4.2 Redox control of T-cell bioenergetics: insights into immunometabolism

The role of ROS in T-cell biology is multifaceted, extending beyond their traditional association with oxidative stress. TCR signaling intricately orchestrates an array of cellular responses, including the activation of key transcription factors such as NF-κB, NFAT1, and AP-1, ultimately leading to an upsurge in ROS production from mitochondria and NOXs (5, 63). This surge in ROS emerges as a critical player in signaling pathways essential for T-cell activation, differentiation, and proliferation demand a significant metabolic reprogramming (63, 82). The physiological functions of ROS extend to acting as intracellular messengers, influencing the activity of signaling proteins like p53, c-Jun, Fos, and NF-kB subunits (5, 83). Long-lived ROS also partakes in intercellular communication, affecting neighboring cells. Immune cell activation, intricately linked with specific metabolic pathways, involves a redox system rearrangement regulated by mammalian target of Rapamycin (mTOR), Phosphoinositide 3-kinase (PI3K)/Protein kinase B (AKT), Hypoxia inducible factor 1α (HIF-1α), and c-Myc (63, 84).

Distinct T-cell subsets exhibit unique metabolic demands. Effector functions and the differentiation of CD4^+^ and CD8^+^ T-cells heavily rely on aerobic glycolysis and the pentose phosphate pathway (PPP), regulated by HIF-1α and mTOR (85). In contrast, naive and memory T-cells, as well as Tregs, preferentially utilize oxidative phosphorylation (OXPHOS) and fatty acid oxidation (FAO) to meet their modest metabolic needs (86, 87). Notable differences in fatty acid metabolism between effector and memory T-cells underscore the intricacies of T-cell metabolism (63, 88). TCR signaling also modulates amino acid transporters, impacting glutamine catabolism and amino acid uptake crucial for T-cell survival. The observations presented here strongly suggest that ROS in the GI tract possesses the capacity to influence the metabolic programming of T-cells. This influence may play a pivotal role in shaping various aspects of T-cell function, highlighting the complex and interdependent relationship between ROS and T-cell metabolism in the GI tract (6, 89).

### 4.3 Redox signaling in T-cell differentiation

The regulation of T-cell differentiation, proliferation, and survival hinges on the intricate interplay between ROS and cellular antioxidant pathways. This dynamic equilibrium is orchestrated by key players such as thioredoxin (TRX) and GSH, under the control of the transcriptional regulator NRF-2 (88). Within the T-cell microenvironment, an optimal redox balance, characterized by low levels of ROS, favors Th1 and Th17 polarizations. Conversely, an excess of ROS promotes a Th2 polarized phenotype (63, 90). Compromised antioxidant defenses leading to elevated ROS levels can have detrimental consequences, impacting mitochondrial membrane polarization and jeopardizing T-cell activation and survival, as illustrated in **Figure 3** (91). The chronic elevation of ROS levels poses the risk of T-cell hyperresponsiveness, exhaustion, and energy depletion (92). Safeguards against harmful ROS accumulation are provided by antioxidant defense systems, including glutathione and thioredoxin/thioredoxin reductase (TRX/TRXR) (93). TCR activation triggers GSH synthesis, influencing T-cell survival and function, while chronic nitro-oxidative stress depletes GSH, compromising metabolic reprogramming and sustaining aerobic glycolysis.

**Figure 4:**
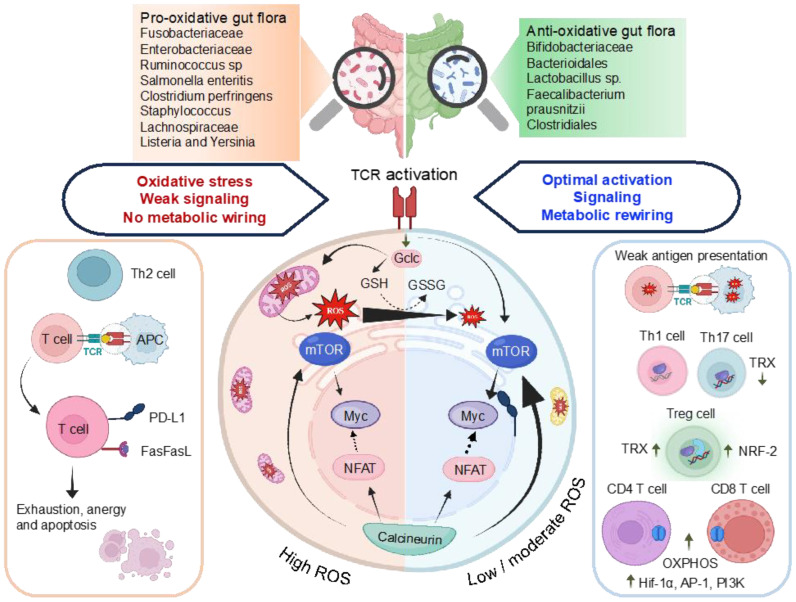
Illustration of the interlinked association among ROS, antioxidant characteristics of gut microbiomes, and the control of transcriptional regulators, metabolic reprogramming, and T-cell activation. TCR stimulation triggers mTOR activation and a concurrent increase in ROS levels. Subsequent T-cell activation induces the expression of Gclc, facilitating the production of GSH. The regulation of ROS through GSH buffering activity, as indicated by the GSH to GSSG ratio, preserves the integrity of T-cell energy dynamics and metabolic restructuring upon antigen-induced activation. This ensures the efficient activation of mTOR and Myc, enabling the T-cell to generate a functional response. Chronic ROS stress depletes GSH, compromising mTOR, NFAT, and N-Myc function. High ROS promote Th2, inhibit NFAT5 decrease T-cell proliferation, upregulate PD-L1, trigger activation-induced cell death of T-cells via Fas/FasL pathway. Low ROS weak antigen presentation, favor Th1 and Th17 polarizations. High TRX-1 production influences, fostering the generation of Tregs from naive T-cells and reducing differentiation toward Th1 and Th17 pathways. High levels of HIF-1α and mTOR enhance OXPHOS, pentose phosphate pathway (PPP), Fatty acid (FA) synthesis and glutaminolysis. The effector functions and differentiation of CD4^+^ and CD8^+^ T-cells demand elevated OXPHOS activity. Gclc, glutamate-cysteine ligase catalytic subunit is involved in OXPHOS, oxidative phosphorylation; FA synthesis, PPP, GSH, glutathione; GSSG, glutathione disulfide and HIF1α, hypoxia-inducible factor 1-alpha.

Tregs influence effector T-cells by modulating GSH synthesis, contributing to immune homeostasis. The transcriptional regulator NRF-2 plays a crucial role in fine-tuning the redox balance to modulate T-cell immunity. Studies indicate that overexpression of NRF-2 amplify Treg proliferation, enhancing their immunosuppressive functions. NRF-2 downregulation increases sensitivity to Fas-mediated apoptosis by regulating intracellular GSH levels (**Figure 4**). Tregs employ ROS as a signaling molecule to regulate immune responses, utilizing it to suppress other immune cells and maintain immune tolerance. The TRX system, particularly TRX-1, influences T-cell proliferation and activation, hindering stimulation and promoting Treg generation (88). Upregulated TRX-1 is essential for sustaining the survival and function of T-effector and Treg cells during chronic nitro-oxidative stress, safeguarding membrane protein thiols from oxidation. Elevated TRX-1 activity is also crucial for maintaining IL-2 production. This intricate web of redox regulation in T-cell biology highlights the multifaceted nature of ROS in shaping immune responses and maintaining immune homeostasis (**Figure 4**).

### 4.4 Redox signaling orchestrating T-cell effector functions

The dynamic interplay of redox signaling within T-cells intricately governs their effector functions, ensuring a finely tuned response to immune challenges. The fundamental regulatory role of ROS in cytotoxicity, cytokine production, and T-cell differentiation is paramount for the effective execution of immune responses. Specifically, cytotoxic T-lymphocytes (CTLs) depend on ROS for cytotoxicity, where molecules like butyrate enhance CTL activation and the secretion of critical molecules, including IFN-γ and granzyme B. In the intestinal environment, microbiota-derived SCFAs further shape the response of CD8^+^ T-cells. The generation of ROS by cytotoxic T-cells is indispensable during immune responses for targeting infected or transformed cells. Crucially, mitochondrial ROS emerges as a modulator of T-cell activation, influencing the secretion of vital cytokines such as IL-2 and IL-4. NOX-2 derived ROS play a pivotal role in enhancing IFN-γ production through intricate mechanisms involving the activation of signaling pathways, including c-Jun N-terminal kinase (JNK), signal transducer and activator of transcription 1 (STAT-1), NF-κB, and T-bet. Additionally, mitochondrial ROS exerts regulatory control over the differentiation of specific T-cell subsets, notably Th17 cells and Th1 cells. Low levels of ROS contribute to the induction of indoleamine 2,3-dioxygenase, playing a role in the immunoregulatory functions of Tregs. The NOX/ROS system emerges as a critical upstream component in signaling pathways crucial for the development of cytotoxic CD8^+^ T-cells. In summary, redox signaling stands out as a central orchestrator, shaping T-cell effector functions and influencing cytotoxicity, cytokine production, and the differentiation of T-cell subsets in response to diverse immune challenges (**Table 3**).

**Table 3:** List of oxidants and antioxidants affecting T-cell functions.

Redox mechanism	T-cell function	References
Reactive oxygen species (ROS)	Antigen presentation, T-cell activation, Regulate Th1/Th2 balance, Favor Th2 polarization, Dysregulate T-cell homeostasis	(60, 69, 70, 90, 92, 94)
Glutathione (GSH)	Reducing oxidative stress, Maintaining T-cell redox balance, Suppresses Th17 differentiation, Encourages the production of Tregs	(7, 95–97)
S-S-Transferase (GST)	Involved in detoxification, modulates intracellular oxidative stress, Protecting T-cells	(3, 98)
Glutamate-cysteine ligase catalytic (GLLC)	Glutathione biosynthesis, Involved in cellular antioxidant defense mechanism, Increased glutathione levels, Support T-cell survival and proliferation	(99–101)
Thioredoxin (TRX)	Serve as an antioxidant protein, Protecting T-cells from oxidative damage, Induces IL-2R expression on T-cells, Overexpression blocks NF-κB activation by H_2_O_2_	(22, 102)
N-acetyl cysteine (NAC) and apigenin	Act as Antioxidant, inhibit antigen presentation and PD-L1 expression by reducing ROS levels	(72)
Vitamins (C and E)	Reducing oxidative stress, Improve T-cell health and function.	(60, 103, 104)

## 5 GM-mediated redox homeostasis: direct effects on T-cells

The dynamic interplay between the gut microbiome and redox equilibrium within the GI tract constitutes a multifaceted regulatory system influencing host immune responses. This intricate relationship is characterized by a delicate balance between ROS generation and antioxidant activities orchestrated by the microbiota (63). Commensal and pathogenic bacteria play pivotal roles in modulating ROS production, impacting mitochondrial function, and activating NADPH oxidases. Moreover, the microbiome exerts direct control over T-cell activity by regulating nutrient utilization, influencing host metabolism, and modulating the production of essential vitamins and enzymes.

Diverse metabolites generated by the microbiota, such as SCFAs, ethanol, lactate, phenols, and succinate, along with the transformation of bile acids, contribute to the regulation of redox homeostasis in the gut (105). This microbial influence extends beyond metabolic pathways, encompassing degradation of proteins and carbohydrates. However, the dynamic roles of the gut microbiota are not limited to metabolic processes; they extend to vital immune regulatory functions. The microbiome efficiently limits the growth of harmful bacteria in the GI tract, sustaining a harmonized immune environment (5, 84).

**Figure 5:**
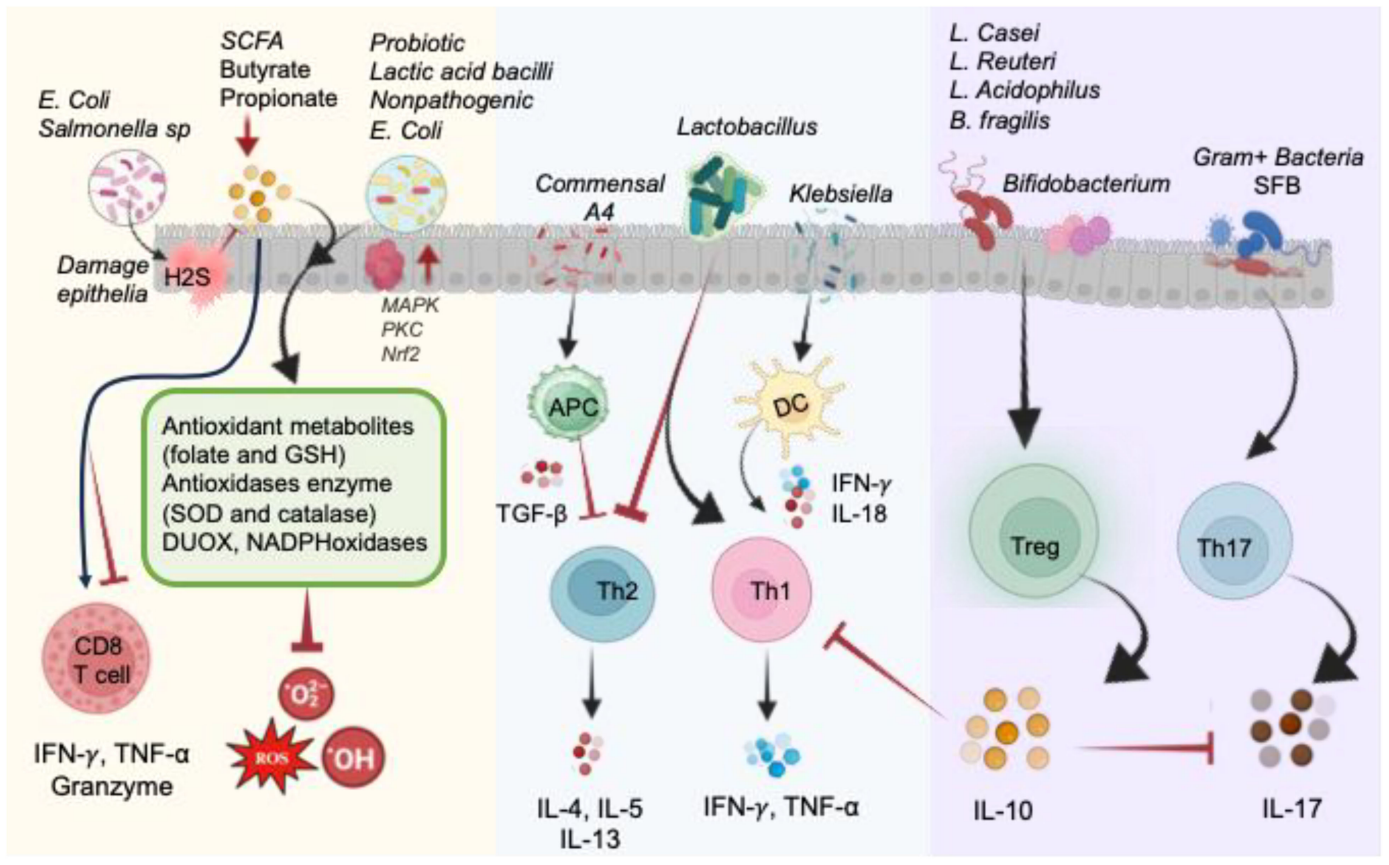
Illustration of direct and indirect impact of the gut microbiome on the redox status of the intestine in normal physiological conditions. Pathogenic bacteria like *E. coli* and *Salmonella* produce H_2_S, causing damage to epithelial cells by blocking complex IV of the electron transport chain and hindering SCFA metabolism. Conversely, probiotic commensals generate antioxidant metabolites (folate and GSH) and antioxidant enzymes (SOD and CAT), as well as DUOX and NADPH oxidases, to reduce oxidized molecules and mitigate ROS. These actions are facilitated by MAPK, PKC, and NRF2 signaling pathways. Microbial byproducts, particularly butyrate and propionate, impact CD8^+^ T-cell function. Lactobacillus species promote Th1 cells and inhibit Th2 cells. Commensal A4 inhibits Th2 cells by enhancing TGF-β-secreting APC. *Klebsiella*-induced Th1 cell differentiation occurs via Batf3-dependent dendritic cells and IL-18 signaling. Gram-positive bacteria, such as segmented filamentous bacteria (SFB), play a crucial role in inducing Th17 cell differentiation. *Bacteroides fragilis* and *Lactobacillus* species promote the development of IL-10-secreting Treg cells by producing polysaccharides. Probiotics contribute to restoring the equilibrium of immune responses regulated by Th17 and Treg cells.

The involvement of pathogenic bacteria, such as *E. coli* and *Salmonella*, introduces the complexity of hydrogen sulfide (H_2_S) generation during the degradation of sulfur-containing amino acids. Excessive H_2_S levels impair colonic epithelial cell functions, resulting in epithelial damage, disruption of the mucus barrier, and interference with SCFAs metabolism (106). On the contrary, commensal bacterial communities, particularly probiotics, emerge as key contributors to redox homeostasis (5, 107). Probiotics exhibit antioxidative properties through the production of antioxidizes, such as SOD and CAT, and the generation of antioxidant metabolites like folate and GSH (108, 109). These beneficial microorganisms also induce antioxidative capacities in host cells, while simultaneously dampening the activities of ROS-producing enzymes. These host gut health supporting include lactic acid bacilli, *E. coli Nissle* 1917, *Saccharomyces boulardii*, and others (108, 110). The impact of the gut microbiota extends to immune cell function and differentiation, influencing the development of CD4^+^ and CD8^+^ T-cells. Recent studies highlight the microbiome’s role as an antigen, shaping microbiota-specific T-cell development in the thymus (101, 111). The differentiation of T-cells into effector Th1, Th2, Th17, regulatory Treg cells, and cytotoxic CD8^+^ T-cells is modulated by distinct commensal microbiota, contributing to the maintenance of host immune balance (**Figure 5**).

### 5.1 Impact on CD8^+^ T-cell regulation

CD8^+^ T-cells emerge as pivotal players in the immune defense against intracellular pathogens, underscoring their significance in combatting viral and bacterial infections. Their multifaceted role extends beyond infectious diseases, encompassing the pathogenesis of inflammatory bowel diseases. The modulation of CD8^+^ T-cell phenotype and function by gastrointestinal commensals adds another layer of complexity to our understanding of immune regulation. Notably, certain probiotic species, such *as Mobilicoccus massiliensis* and *Bifidobacterium*-derived pentanoate, demonstrate the ability to activate CD8^+^ T-cells, enhancing anti-tumor responses (101, 112). Commensals like *B. fragilis, B. thetaiotaomicron*, and *Burkholderiales* contribute to the effectiveness of cancer immunotherapy, as illustrated by Vétizou et al. (113). The influence of microbial byproducts, specifically butyrate, and propionate, on CD8^+^ T-cell function highlights the intricate balance maintained by the gut microbiota. The dichotomy observed in the effects of butyrate, wherein it can both inhibit and enhance CD8^+^ T-cell activity, exemplifies the nuanced relationship between microbial metabolites and immune responses. Moreover, specific bacterial strain-derived metabolites, such as mevalonate, dimethylglycine, and SCFAs, demonstrate the capacity to systemically activate CD8^+^ T-cells (101). These findings collectively shed light on the intricate interplay between CD8^+^ T-cells and various microbial factors, showcasing their paramount importance in cancer, autoimmunity, and gut health (114, 115). Understanding these interactions provides valuable insights into inflammatory responses and immunopathology, offering potential avenues for therapeutic interventions in diverse clinical contexts. The dynamic relationship between CD8^+^ T-cells and the microbiome underscores the need for further research to unravel the complexities of this interdependent network and harness its therapeutic potential.

### 5.2 Direct effect on CD4^+^ T-cell regulation

#### 5.2.1 Th1 and Th2 cells

The gut microbiota profoundly influences CD4^+^ T-cell behavior, modulating subsets like Th1, Th2, Th17, and Treg cells. *Klebsiella* genera trigger Th1 responses by stimulating APCs to secrete IFN-*γ* and TNF-α, promoting Th1 cell proliferation in GF mice (116). This process involves Batf3-dependent dendritic cells and TLR signaling with IL-18 (116). *Klebsiella* dominance during dysbiosis may induce Th1 cell differentiation, contributing to gut inflammation observed in IBD patients (116, 117). Conversely, *Lactobacillus* strains like *L. plantarum and L. salivarius* can influence Th1 cell activity, enhancing TNF-α and IFN-*γ* production (118, 119). *Lactobacillus* isolated from fermented foods also boosts TNF-α secretion via macrophage activation while reducing IL-4 levels (120). *Lactobacillus* strains and *B. fragilis* were identified as inhibitors of Th2 activity while promoting Th1 activity (118, 119, 121). Th2 cells, characterized by their secretion of interleukins IL-4, IL-5, and IL-13, play a crucial role in humoral immunity, defense against helminth infections, and contribute to chronic inflammatory diseases. The intricate interplay between Th2 cells and the microbiota further underscores the complexity of immune regulation. *Lactobacillus* strains and *B. fragilis* have been identified as modulators of Th2 activity, positively influencing Th1 responses (118, 119, 121) (**Figure 5**). This bidirectional regulation sheds light on the dynamic nature of the host-microbiota interaction. The influence of the microbiota on Th2 activity becomes particularly evident in the context of IBD, as demonstrated in SAMP1/YitFc mouse models of Crohn’s disease-like ileitis (122). Commensal bacteria induced Th2 responses during the chronic phase exacerbate symptoms, emphasizing the pivotal role of microbial factors in shaping the immune landscape in chronic inflammatory conditions.

#### 5.2.2 Direct modulation of Th17

Furthermore, commensal bacteria prove to be essential in balancing pro- and anti-inflammatory cytokine production by Th17 and Treg cells. The IL-17-producing Th17 cell subset represents a highly proinflammatory component of CD4^+^ T-cells, implicated in tissue damage and recognized as a key player in the pathogenesis of various inflammatory diseases (123). The influence of the gut microbiome on Th17 cell differentiation and function is a critical aspect observed in autoimmune conditions such as multiple sclerosis (MS), rheumatoid arthritis (RA), and IBD (101, 124). Gram-positive bacteria, particularly segmented filamentous bacteria (SFB), emerge as crucial contributors to Th17 cell induction. The differentiation of Th17 cells is intricately linked to the activation of lamina propria cells by adenosine triphosphate (ATP) derived from SFB or other commensal bacteria (116, 125). Noteworthy examples include *‘Candidatus Arthromitus’* (SFB) and Prevotella, both gram-positive bacteria, stimulating Th17 cell differentiation and promoting the secretion of IL-17 and IL-22 in the mouse colon. The specificity of certain bacteria in Th17 cell differentiation is highlighted by studies utilizing a gram-positive bacterial antibiotic, vancomycin. Administration of vancomycin to specific pathogen-free (SPF) mice results in a reduction of Th17 cells in the small intestine, emphasizing the targeted impact of particular bacterial strains on this T-cell subset (126). These findings underscore the intricate relationship between the gut microbiome and Th17 cells, shedding light on the role of specific bacteria in shaping the proinflammatory response associated with Th17 cells (127). The modulation of Th17 cell activity by the gut microbiome presents potential therapeutic avenues for managing inflammatory diseases, emphasizing the importance of understanding, and targeting the microbiome-immune axis in the development of novel treatment strategies. As research in this field continues, unraveling the precise mechanisms governing Th17 cell modulation by the microbiota holds promise for advancing our understanding of autoimmune diseases and informing future therapeutic interventions.

#### 5.2.3 Direct modulation of Treg

Foxp3^+^ Treg cells act as crucial regulators, preventing unwarranted immune responses against dietary antigens and commensal bacteria. Their deficiency has been implicated in dysbiosis, emphasizing their significance in shaping, and preserving a healthy gut microbiota. The Treg-directed establishment of a balanced gut microbiota appears to involve various mechanisms. Foxp3^+^ Treg cells, under certain conditions, differentiate into T-follicular helper (Tfh) cells, which contribute to the production of bacteria-specific IgA in Peyer’s patches (128). This process aids in microbiota diversification and further emphasizes the intricate relationship between the immune system and the gut microbiota The symbiotic association between Foxp3^+^ Treg cells and commensal gut microbes is exemplified by specific microorganisms, such as *Bacteroides fragilis*, *Bifidobacterium*, and various *Lactobacillus* strains (101). These microbes have been shown to induce and activate Treg cells, demonstrating a mutualistic relationship. *B. fragilis*, for instance, stimulates Treg cell populations through the production of polysaccharides (129) (**Figure 5** **).** Similarly, *Bifidobacterium* modulates Treg cells and alleviates inflammation by influencing Th1, Th2, and Th17 cells. The beneficial effects of *Lactobacillus* strains, such as *L. casei, L. reuteri, L. acidophilus* strain L-92, and *L. murinus*, in promoting Foxp3^+^ expression and IL-10 production highlight the diverse impact of commensal bacteria on Treg cell development and function (127, 130, 131). The positive correlation between certain bacterial strains and autoimmune diseases, such as the decreased presence of *Clostridia* strains in rheumatoid arthritis and the beneficial effects of *B. fragilis* in IBD, suggests that the colonization of intestinal Treg cells is associated with specific bacteria and may have implications for immune-related disorders (101). While the exact mechanisms of Treg cell differentiation by specific commensal bacteria remain unclear, the evidence presented underscores the critical role of Foxp3^+^ Treg cells in maintaining immune balance within the gut.

Understanding the complex interplay between CD4^+^ helper and regulatory T-cells and the microbiota provides insights into immune responses in health and disease. Restoring Th17 and Treg cell balance through specific bacteria offers a secondary defense against excessive ROS production in the epithelium. This highlights the broader impact of the microbiota on immune homeostasis and suggests potential therapeutic interventions targeting the microbiome-immune axis. These findings pave the way for developing strategies to modulate the gut microbiota, promoting immune tolerance, and mitigating autoimmune and inflammatory conditions.

## 6 GM-mediated redox homeostasis: indirect effects on T-cells

**Figure 6:**
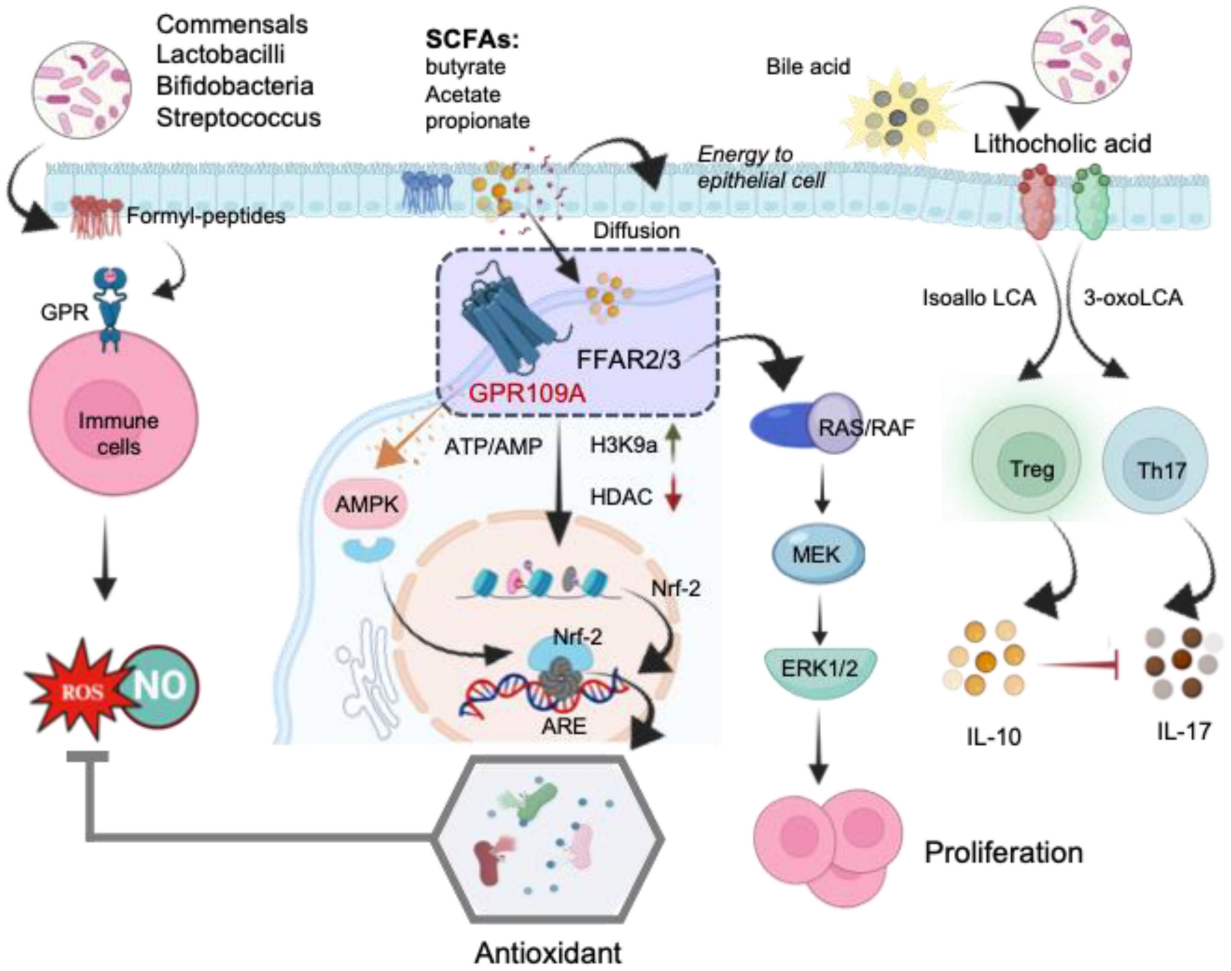
The schematic illustration of the mechanisms underlying the direct regulation of T-cells by microbial metabolites. Formyl peptides trigger the activation of G protein receptors (GPR) on immune cells, leading to ROS generation in the gut. Short-chain fatty acids (SCFAs) bind to free fatty acid receptors (FFARs), activating the RAS/RAF/MEK/ERK signaling pathway and enhancing proliferation. The stimulation of the FFAR receptor by butyrate alters the AMP/ATP ratio, activating AMPK, which subsequently induces the translocation of Nrf-2 into the nucleus. Inhibition of histone deacetylases (HDACs) increases Nrf-2 synthesis, leading to Treg generation. In the nucleus, Nrf-2 binds to the antioxidant response element (ARE), activating the antioxidant defense system. SCFA, short- chain fatty acid; FFAR, free fatty acid receptor; GPR109A, G-protein-coupled receptor; H3K9ac, histone H3 acetylated in lysine 9; RAS, rat sarcoma virus protein; RAF, rapidly accelerated fibrosarcoma protein; MEK, mitogen activated kinase; ERK, extracellular signal regulated kinase; AMPK, AMP-activated protein kinase; HDAC, histone deacetylase; Nrf-2, Nuclear Factor Erythroid 2-related Factor 2.

In addition to directly affecting the production of ROS, antioxidants, and RNS, bacteria employ various strategies to indirectly regulate the redox state and influencing T-cell homeostasis. Metabolites such as formyl-peptides, SCFAs, including butyrate, propionate, and acetate derived from gut microbial fermentation, impact the redox balance. SCFAs play a crucial role in the regulation of T-cell differentiation and function (132). These microbial metabolites impact the activation and function of CD4^+^ and CD8^+^ T-cells through mechanisms involving epigenetic modifications and immune cell signaling. CD4^+^ T-cells, enriched in intestinal tissue, play a key role in maintaining homeostatic responses. Germ-free animal studies indicate an imbalance between Th1 and Th2 immune responses, favoring a Th2 type of immune response. The metabolic reprogramming of naïve CD4^+^ T-cells leads to the development of unique subsets, such as Th17 and Treg cells, which play a role in managing pathologies that can harm the host (133). Formylated peptides derived from commensals bind to G protein receptors (GPR) on immune cells, including macrophages, neutrophils, and epithelial cells, triggering inflammatory processes and increasing ROS generation in the gut through the activation of NADPH oxidases (5, 134) (**Figure 6**). Additionally, certain bacteria like *Lactobacilli, Bifidobacteria, Streptococcus*, and *Bacilli* have the capability to synthesize NO (17, 135). These SCFAs play diverse roles in T-cell regulation in addition to serving as an energy source for epithelial cells influencing neuronal development and various physiological functions of organs through systemic circulation (5).

The regulatory actions of SCFAs rely on specific receptors expressed in various cell types. Research indicates that free fatty acid receptors (FFARs) play a role in regulating metabolic energy and influencing the immune system (136). Butyrate’s interaction with the FFAR receptor modifies the AMP/ATP ratio, activating AMP-activated protein kinase (AMPK). This activated AMPK facilitates the translocation of Nrf-2 into the nucleus. Nrf-2, a pivotal transcription factor, controls over 200 genes involved in cellular antioxidant defense (137) (**Figure 6**). In the nucleus, Nrf-2 binds with antioxidant response element (ARE) to activate the antioxidant defense system. Moreover, SCFAs activate FFARs, initiating the RAS/RAF/MEK/ERK signaling pathway, leading to proliferation (136). Additionally, inhibiting Histone deacetylases (HDACs) enhances the synthesis of Nrf-2, thus activating oxidative defense mechanisms. Among the SCFAs, butyrate stands out as the most potent inhibitor of HDAC activities. Mechanistically, butyrate hinders the recruitment of HDACs to the promoter by transcription factors specificity protein 1 and 3 (Sp1/Sp3), resulting in histone hyperacetylation (138). Through HDAC inhibition, butyrate plays a pivotal role in suppressing cell proliferation, differentiation, and apoptosis in T-cells. It also contributes to the downregulation of proinflammatory effectors in lamina propria macrophages and regulates cytokine expression in T-cells (139). Collectively, these studies highlight epigenetic regulation, involving HDACs and/or Nrf-2 nuclear translocation, as the main reported mechanisms of action for butyrate. By strengthening antioxidant defenses, SCFAs mitigate ROS mediated mitochondrial damage and enhance mitochondrial function. This protective action not only safeguards mitochondrial metabolism but also ensures a more efficient energy supply to the cells.

Butyrate and propionate have been reported to increase colonic FoxP3^+^ Tregs, potentially through their HDAC inhibitor activity (140–142). *Clostridia* induce colonic peripherally induce Treg (pTreg) cells through butyrate production, and their oral administration to mice imparts resistance to colitis and allergic diarrhea (143–145). Additionally, all major SCFAs, including acetate, propionate, and butyrate, enhance the generation of effector T-cells (Th17 and Th1) as well as IL-10^+^ CD4^+^ T-cells in both *in vitro* and *in vivo* settings (146, 147). In a mouse model study, SCFAs, specifically butyrate produced during starch fermentation by commensal microorganisms, were found to facilitate the extrathymic generation of Treg cells. The increased Treg-cell numbers induced by butyrate were attributed to the potentiation of extrathymic differentiation, dependent on the intronic enhancer CNS1 (141). Experimental studies have shown that butyrate enhances extrathymic differentiation of Treg cells. These studies utilized Specific Pathogen-Free (SPF) mice lacking an intronic Foxp3 enhancer CNS1, resulting in selective impairment of extrathymic Treg cell differentiation while maintaining intact thymic differentiation. Findings of this study revealed that butyrate was unable to restore impaired Foxp3 induction in naïve CD4^+^ T cells lacking CNS1. Additionally, extracts of feces from SPF mice facilitated Foxp3 induction when peripheral naïve CD4^+^ T cells were stimulated with CD3 antibody in the presence of DCs, IL-2, and TGF-β. Furthermore, *in vitro* experiments demonstrated that butyrate treatment increased the number of Foxp3^+^ cells in DC-free cultures of purified naïve CD4^+^ T cells stimulated by CD3 and CD28 antibody-coated beads and TGF-β. This effect was likely attributed to enhanced Foxp3 protein acetylation induced by butyrate, a known histone deacetylase (HDAC) inhibitor (148, 149). Another SCFA, propionate, with HDAC inhibitory activity, also enhanced *de novo* Treg-cell generation in the periphery (141). In contrast, acetate, which lacks HDAC-inhibitory activity, did not demonstrate a similar effect (141). These observations underscore the significance of bacterial metabolites in facilitating communication between the commensal microbiota and the immune system, thereby impacting the equilibrium between pro- and anti-inflammatory mechanisms. Commensal microbe-derived butyrate has been shown to induce the differentiation of colonic regulatory T-cells and inhibit IL-17, generating Tregs to alleviate colorectal colitis in rats (149, 150). Additionally, bile acid metabolites from gut microbes promote Treg cell differentiation through the generation of mitochondrial ROS (151).

Bile acid metabolites derived from gut bacteria exhibit suppressive effects on Th17 cells and promote the activation of CXCR5^+^ CD4^+^ T follicular helper (Tfh) cells in Neuromyelitis Optica Spectrum Disorder recurrence (152). Furthermore, bile acid metabolites contribute to the differentiation of Th17 and Treg cells (151). Hang et al. recently identified two derivatives of lithocholic acid (LCA), 3-oxoLCA and isoalloLCA, as T-cell regulators (**Figure 6**). Simultaneous administration of these derivatives decreased Th17 cell differentiation and elevated Treg cell expression in the ileum. The suppressive impact of 3-oxoLCA on Th17 cells was facilitated by ROR*γ*t inhibition, whereas isoalloLCA boosted Treg cells by increasing mitochondrial reactive oxygen species production and enhancing FOXP3^+^ expression through the intronic enhancer conserved noncoding sequence (CNS). Additionally, 3-OxoLCA strengthened the activity of isoalloLCA on Treg cells (151). This data unveils a novel mechanism through which bile acid metabolites directly modulate the balance between Th17 and Treg cells, influencing host immune responses. The primary gut microbiota species responsible for converting lithocholic acid (LCA) to 3-oxoLCA and isoLCA include *Bifidobacterium, Enterocloster, Adlercreutzia, Clostridium, Collinsella, Eggerthella, Gordonibacter, Mediterraneibacte, Monoglobus, Peptoniphilus, Phocea*, and *Raoultibacter* (153). Overall, SCFAs significantly impact regulatory T-cells and effector T-cells based on the immunological context, with intestinal T-cells being a major target due to elevated SCFAs levels in colonic tissues and gut-associated lymphoid tissues.

## 7 TRM cell regulation

The GI tract, being the largest mucosal surface, faces threats from pathogens. It boasts various defense mechanisms against pathogen invasion and tumor formation, including a network of innate and adaptive immune cells. This immune response is crucial as the GI tract interacts with the gut microbiome and absorbs nutrients. Immunological memory, particularly in naïve CD8^+^ T-cells, plays a pivotal role. Upon infection, these cells rapidly expand to mount an effective defense, ensuring long-lasting immunity. Generation of memory T-cells are a critical component of the adaptive immune system that provides long-lasting immunity against previously encountered pathogens. These memory T-cells can be broadly categorized into three primary populations: central memory T-cells (TCM), effector memory T-cells (TEM), and tissue-resident memory T-cells (TRM), distinguished by their location, function, and migratory properties (154, 155). TRM cells are an integral part of the immune system’s defense strategy, persisting in various locations throughout the body demonstrating their versatility in immune surveillance and response. While they are prominently found in barrier tissues like the GI tract, skin, and lungs, they also reside in non-barrier tissues such as brain (156–158). The GI tract is constantly exposed to various commensals, pathogenic bacteria, viruses, and other potentially toxic agents. Within this environment, the balance of redox reactions, microbiota-derived compounds, and metabolites profoundly affects various aspects of the host’s physiological functions, particularly the response of TRM cells. These cells, crucial for immune defense, are in close proximity to the gut microbiota within the mucosal barrier. Environmental factors such as dietary patterns and nutritional status significantly influence the intricate relationship between the host and its gut microbiota, impacting immune responses. The signals generated from this dynamic interaction, along with the production of metabolites, and ROS likely play a pivotal role in shaping the development, maintenance, and functionality of TRM cells within the GI track.

**Figure 7:**
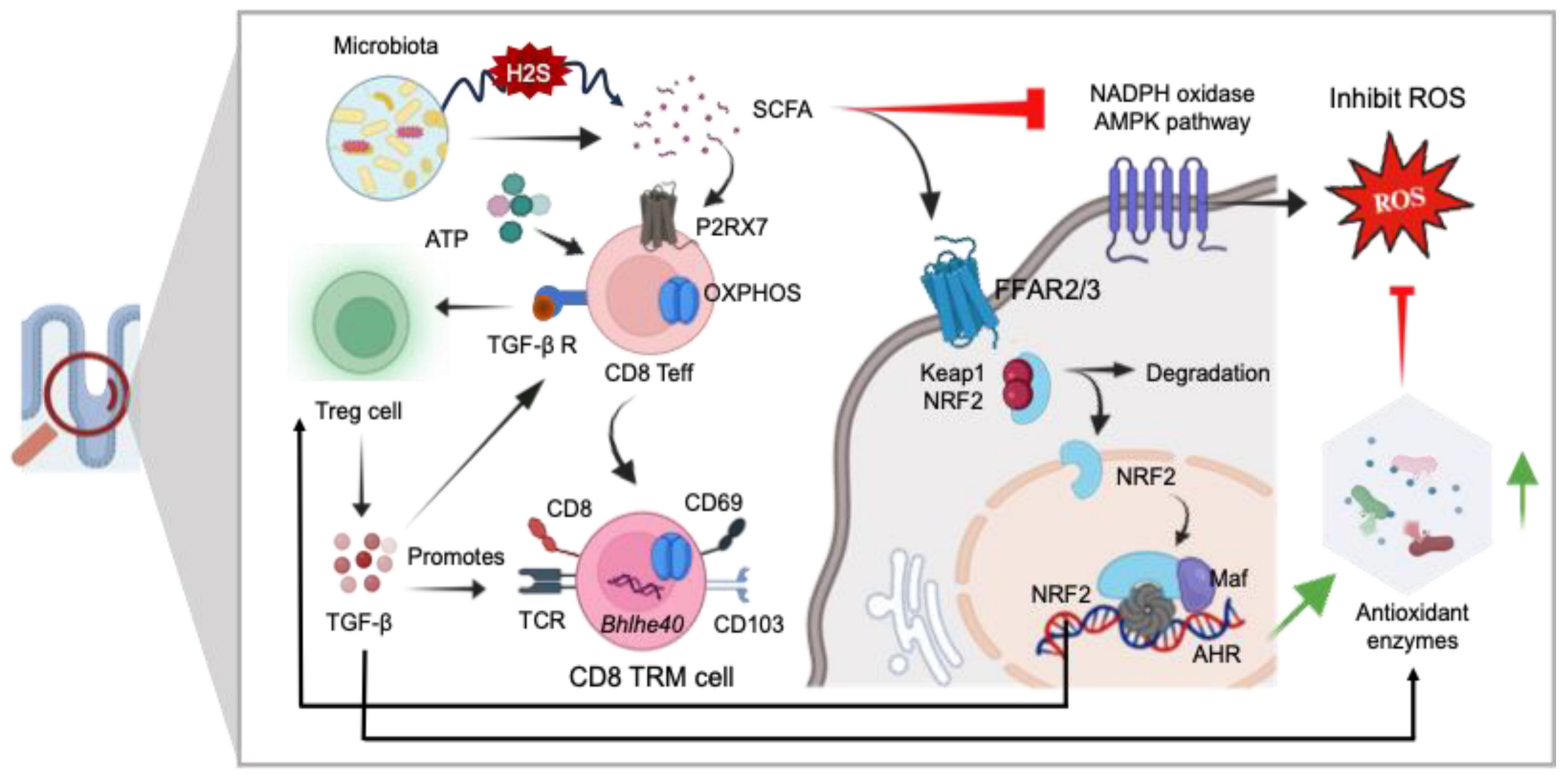
The role of GI redox homeostasis and TRM cell regulation. SCFAs exhibit potent anti-inflammatory effects and serve as modulators for the differentiation of CD8^+^ effector T-cells (Teff) into CD8 Tissue-Resident Memory (TRM) cells *via* P2RX7 signaling mediated OXPHOS. SCFAs also regulate cellular redox homeostasis by activating signaling pathways involving Nrf-2, a master regulator of antioxidant defense mechanisms (SOD, GSH, CAT, and GPx), and directly inhibiting the NADPH oxidase AMPK pathways. Additionally, SCFAs contribute to the stabilization of gut resident Treg cells, thereby promoting the generation of CD8^+^ TRM cells. Nrf-2 exerts antioxidant activity in the presence of ROS. Keap1, Kelch-like ECH-associated protein 1; ARE, antioxidant response element; SOD, superoxide dismutase; GSH, glutathione; CAT, catalase; GPX, glutathione peroxidase.

Studies indicate the involvement of the adenosine monophosphate-activated protein kinase (AMPK)/nicotinamide adenine dinucleotide phosphate (NADPH) oxidase signaling pathway in oxidative stress and inflammatory responses (159). There is growing evidence that metabolite produced by gut microbiota plays a role in regulating AMPK activity (160). SCFAs reduced the activating enzymes like NADPH oxidases within the host cells, which are responsible for ROS generation (136) (**Figure 7**). Accumulating evidence indicates that SCFAs mediate significant anti-inflammatory action by restricting ROS production which may prevent excessive immune response and maintaining gut epithelial barrier (161, 162). The SCFAs not only stabilize gut-resident regulatory T-cells and diminish inflammatory innate immune cells but also enhance the survival and function of CD8^+^ memory T-cells (141, 163). Gut microbiota-derived SCFAs direct T-cell metabolism toward OXPHOS and Fatty Acid Oxidation (FAO), benefiting long-term survival (164). Additionally, SCFAs influence the capacity of antigen-activated CD8^+^ T-cells to differentiate into memory CD8^+^ T-cells with enhanced recall response (165). Additionally, it is well established that SCFAs acts as a modulator for cellular redox homeostasis by inducing a signaling pathways, involving Nrf-2 a master regulator of antioxidant defense (136, 137). In normal circumstances, Nrf-2 is confined within the cytoplasm by Keap1, which acts as a substrate adapter protein, aiding in the ubiquitination and subsequent degradation of Nrf-2 through the proteasome. However, under oxidative stress conditions, Nrf2 is dissociated from Keap1 due to the thiol modification of Keap1 cysteine residues. This allows Nrf-2 to translocate into the nucleus, where it forms a heterodimer with small Maf proteins and binds to the ARE in the promoter regions of target genes. This binding activates the transcription of a wide array of genes encoding detoxifying enzymes, antioxidant proteins, and other cytoprotective factors.

Bacterial pathogens like *Salmonella* and *E. coli* can generate H_2_S while breaking down sulfur-containing amino acids. Elevated levels of H_2_S can disrupt cellular functions, particularly in colonic epithelial cells. Increased levels of H_2_S can interfere with the proper functioning of colonic epithelial cells. H_2_S can interfere with the mitochondrial electron transport chain, specifically blocking complex IV. This interference can impair cellular energy production and overall cellular health. H_2_S induced disruption can affect the metabolism of SCFAs, which are crucial for gut health and various metabolic processes in the body including the long-term survival of T-cells. H_2_S at heightened levels can compromise the integrity of the mucus barrier in the colon (5). The activation of REDOX-sensitive transcription factors like NF-κB and AP-1 is intricately linked to oxidative signaling pathways. When triggered, these transcription factors respond to changes in the cell’s redox environment. It has been implicated that extracellular cues at the time of pathogen challenge are crucial for cell differentiation into either T effector cells (Teff) or Treg cells. Treg cells primarily utilize FAO and OXPHOS as opposed to Teff that use aerobic glycolysis under proinflammatory conditions or increased ROS conditions. Enhanced Treg cell activity has been reported to promote TRM cell maintenance and development *via* TGF-β production. Additionally, TGF-β-activated kinase 1 (TAK1) is considered a key regulator of ROS in the intestinal epithelium. This study demonstrates that the deficiency of TAK1 led to a decrease in the expression of multiple antioxidant-responsive genes and diminished the protein levels of the pivotal antioxidant transcription factor Nrf-2. Studies using mouse models with genetic ablation of TAK1 have demonstrated its essential role in preventing oxidative tissue injury in the epidermis and intestinal epithelium. Deletion of TAK1 specifically in intestinal epithelial cells leads to a decrease in the protein levels of NRF-2 in the intestine. Furthermore, Tak1 deficiency results in the spontaneous upregulation of ROS in these tissues even without external stimulation, indicating that TAK1 plays a crucial role in regulating cellular antioxidant levels under steady-state conditions (166). Consequently, this deficiency resulted in the accumulation of ROS. Moreover, like Treg cells, the development and survival of TRM cells are majorly dependent on OXPHOS (167). When ROS production exceeds the capacity of the cell’s antioxidant systems it impairs OXPHOS complex and further promotes ROS production (168, 169).

A group of transmembrane proteins widely expressed on various immune cells immune cells including CD8^+^ T-cells in the gut (170). Heiss et al. were the first to propose the involvement of eATP/P2X7R in altering intestinal T-cell immunity. P2XR7 plays an important role in purinergic signaling pathway during inflammatory responses (171). At lower ATP concentrations, P2X7R functions as ion channels for Na^+^, K^+^, or Ca2^+^. However, in high ATP environments, P2X7R can create large pores, leading to heightened permeability and triggering cell apoptosis (171). Studies also indicate that when there’s a high presence of extracellular NAD^+^, P2RX7 receptors can facilitate cell death of the gut TRM cells (172). P2RX7 is known to mediate overall mitochondrial homeostasis in the event of low intermittent levels of eATP that induces cytosolic Ca2^+^ influx. Furthermore, recent research suggests that the purinergic receptor P2RX7 plays a pivotal role in the differentiation and maintenance of CD8^+^ TRM cells. Parabiosis experiments involving the conjoining of mice that had received co-adoptive transfer of WT and P2RX7^−/−^ P14 cells, followed by LCMV infection, along with non-transferred, infection-matched controls, indicate that P2RX7^−/−^ P14 cells not only exhibited deficiencies in forming TRM cells but also displayed reduced retention in the tissues. Moreover, P2RX7^−/−^ P14 cells in the small intestine demonstrated higher percentages of cell death at late memory time points. P2RX7 stimulates the expression of TGF-β receptors on effector T-cells, increasing their sensitivity to the cytokine TGF-β and facilitating their differentiation (173). TGF-β triggers a transcriptional program in tissue-infiltrating CD8^+^ T-cells, inducing characteristics akin to TRM cells (174). Additionally, P2RX7 enhances CD103 upregulation on CD8^+^ T-cells which is a known residency marker required interacts with E-cadherin and contributes in the establishment and maintenance of intestinal TRM cells (173, 175). Nonetheless, in patient with Crohn’s disease over expression of P2X7R in intestinal epithelium and lamina propria were found to be linked with increased proinflammatory cytokine, increased rate of apoptosis and lower levels of IL-10 (176). Interestingly, in another study it was suggested that lamina propria Treg cells showed increased rate of apoptosis suggesting impaired immunosuppressive role of Treg cells in chronic IBD (177). Interestingly, another study suggested an increased rate of apoptosis in lamina propria Treg cells, implying a compromised immunosuppressive role in chronic IBD (177). Moreover, research indicates reduced expression levels of CD8^+^CD103^+^ tissue-resident memory cells in the inflamed mucosa of IBD patients. In contrast, ^CD8+^KLRG-1^+^ T-cells, which possess enhanced proliferation and cytotoxic potential, are significantly elevated in these patients (178). These findings underscore the diverse functional profiles of TRM cells within the intestinal mucosa, influencing tissue homeostasis and immunoregulation (178). Purinergic signaling influences the functioning of antioxidant enzyme systems, thereby contributing to alterations in the cellular redox potential. The P2X7 receptor plays a role in regulating the redox potential and can serve as an inducer of H_2_O_2_ production, facilitated by the release of Ca2^+^ (179). P2X7 inhibitors, such as polyphosphonamides and oxidized ATP, effectively hindered H_2_O_2_ production. Conversely, P2X7 agonists like BzATP stimulated the generation of ROS by activating NADPH oxidase in murine macrophages (180). These studies suggest a potential scenario in which elevated levels of P2X7 under inflammatory conditions, along with increased ROS, may play a role in establishing TRM cells. Furthermore, future investigations are essential to comprehend the role of P2X7R in situations characterized by imbalanced ROS.

TRM cell development and maintenance are associated with unique set of transcription factors such Bhlhe40, Blimp-1, Ahr, Runx3, Eomes, and Nur77. Bhlhe40 stands as a stress-responsive transcription factor pivotal in various cellular physiological reactions and responses (167, 181). TGF-β promotes induction of Bhlehe40 in alveolar macrophages and cancer cells (182, 183). Additionally, TAK1 serves as a pivotal regulator of ROS (184). TGF-β also plays a key role in TRM cell development and maintenance, it may be possible that TRM development may be modulated by TGF-β expression under appropriate redox level (167). Moreover, previous studies have reported that Bhlehe40 regulates the function of major organelle involved in the cellular oxidation process. The induction of SOD2 by Bhlehe40 indicates that Bhlehe40 selectively activates ROS scavenging enzymes. In simpler terms, Bhlhe40 plays a crucial role in reducing ROS levels through its mediation of ROS-reducing mechanisms, notably through the activation of SOD2 (185). Additional research is necessary to delve deeper into how Bhlhe40 precisely diminishes ROS levels in the gut. Collectively, a growing body of evidence implicates that redox status of the gut microenvironment influences various cellular activities such as cellular signaling, growth, differentiation, apoptosis, and metabolism. A dense population of memory cells in the human intestinal mucosa are resident in nature that display a distinct phenotype and transcriptional profile contributing to intestinal homeostasis and immunoregulation. These specialized cells generate a robust immune response against reinfection. In recent years, studies have demonstrated that TRM cells participate in the pathogenesis of IBD, other chronic inflammatory responses including intestinal cancer. Therefor TRM might be the potential target to enhance immunological protection.

## 8 MAIT cells regulation

**Figure 8:**
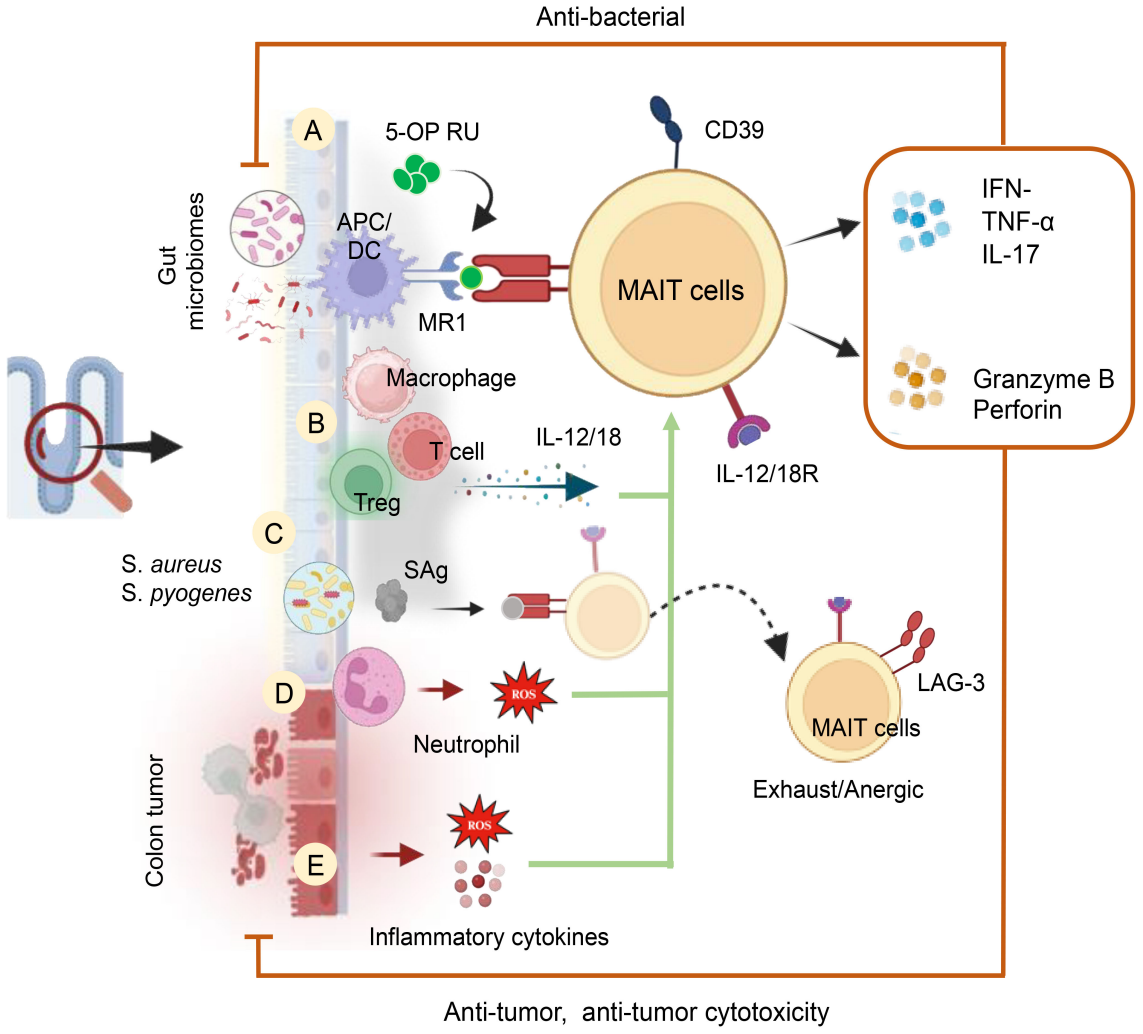
Cellular and Molecular Mechanisms of MAIT Cell Activation in the Human Gut. MAIT cells are activated by **(A)** GM metabolites and a riboflavin biosynthetic pathway in an MR1-dependent manner. **(B)** Activation of MAIT cells occurs through the release of cytokines (IL-12 and IL-18) by inflammatory cells in an MR1-independent manner. These cytokines can be produced by inflammatory macrophages and T-cells. **(C)** MAIT cell activation can also be induced by bacterial-derived super antigens (SAg) in a TCRβ-dependent manner and/or through a cytokine-mediated pathway. Post-cytokine storms upregulate inhibitory receptors like LAG-3, leading to anergy or exhaustion upon subsequent bacterial challenge. **(D)** MAIT cell activation is induced by ROS produced by activated neutrophils and **(E)** Tumor factors, including inflammatory cytokines and ROS, also contribute to their activation.

Mucosal-associated invariant T (MAIT) cells are T-cells with an innate-like nature, featuring a semi-invariant T-cell antigen receptor (TCR) that recognizes microbial non-peptide antigens presented by MR1 (186). Restricted by MR1, MAIT cells recognize bacterial and vitamin B metabolites (187, 188), also activated by cytokines independently of TCR (189). Abundant in mucosal tissues, blood, skin, and liver, constituting 2–5% of human T-cells (190). MAIT cells exhibit potent anti-microbial responses during infections, autoimmunity, and cancers (186, 191). Upon activation, they produce granzyme B, TNF-α, IFN-*γ*, and IL-17A, demonstrating cytotoxicity and inflammatory responses. Additionally, MAIT cells regulate gut epithelial barrier homeostasis (192). Biologically significant markers on MAIT cells encompass the Vα7.2 TCR, co-stimulatory molecules (CD8^+^/^−^, CD4^+^/^−^, CD28), major activation markers (HLA-DR, CD69, CD39, CD38), the coinhibitory molecule LAG-3, and PD-1. Additionally, MAIT cells express various cytokine receptors (IL-12R, IL-15R, IL-18R, and IL-23R), chemokine receptors (CCR5, CCR6, CCR9, CXCR6), as well as CD161, MDR1, CD26, and the degranulation marker CD107a. The key transcription factors expressed by MAIT cells include Eomesodermin, RORγt, PLZF, and Tbet. Like unconventional NKT and γδ T-cells, MAIT cells exhibit swift cytokine responses upon activation, expressing NK-associated CD161 and NKG2D markers, typically linked to NK cells (193). This section explores the influence of ROS and RNS in the GI tract on the differential regulation, activation, and function of MAIT cells, unraveling their interactions with various immune cells in the GI tract and contemplating the prospective therapeutic applications of MAIT cells. The activation of MAIT cells occurs through TCR ligation by riboflavin intermediates presented on MR1, supported by specific cytokines or toll-like receptors (TLR), as illustrated in **Figure 8**. Upon activation, these cells undergo significant expansion, triggering a swift innate-like immune response and demonstrating effector functions, including the generation of anti-microbial cytotoxic products, inflammatory chemokines, and cytokines (194). The microbiota plays a crucial role in the development and activation of MAIT cells. Gut bacteria possessing the riboflavin biosynthesis pathway, such as *Lactobacillus*, *Salmonella*, *E. Coli*, *Staphylococcus*, *Mycobacteria*, *Shigella Flexneri*, and *Clostridioides* species, along with riboflavin-producing fungi like *Aspergillus, Candida and Saccharomyces* can activate MAIT cells in an MR1-dependent manner (194). Among bacterial phyla, Bacteroidetes and Proteobacteria were high stimulators, while Actinobacteria and Firmicutes were less stimulatory. Among MAIT cells Vb2^+^ MAIT cells exhibits the highest activation profiles (195).

Upon activation, MAIT cells identify bacterial antigens, including 5-OP-RU, presented by the MHC class 1b protein MR1, resulting in the release of cytokines such as TNF-α, IFN-γ, and IL-17A (101). These MAIT cells exhibit antimicrobial functions, and infections with *E. coli, Salmonella typhimurium, Francisella tularensis, Mycobacteroides abscessus*, and *Lactococcus lactis* activate and enhance MAIT cell function (101) (**Figure 8**). The gut relies on the presence of neutrophils to balance pathogen protection and prevent excessive inflammation. Dysregulation of neutrophil effect on MAIT cell activation (CD69, OX40) and cytokine production (TNF-α, IFN-*γ*) through release of H_2_O_2_. In an *in vitro* system, the study demonstrated that neutralizing H_2_O_2_ with catalase reversed neutrophil-induced suppression of MAIT cells (196). This insight highlights the intricate interplay between neutrophils and MAIT cells in gut immunity. In colon adenocarcinoma patients, MAIT cells play a significant role, being activated by various bacteria. A recent study by Li et al. (197) explores the impact of MAIT cells on antitumor immune responses in colon cancer, revealing insights into how the microbiota influences MAIT cell modulation. The research indicates an increase in CD39^+^ MAIT cells in colorectal cancer, activated and responsive to microbial antigens in a TCR-dependent manner (197). Notably, gut bacteria associated with CRC, particularly *bacteroides* and *fusobacterium* strains, influence MAIT cell functions. *Fusobacterium nucleatum* and its culture supernatants activate MAIT cells, inducing IFNγ production, dependent on TCR engagement.

The future therapeutic potential of MAIT cells, considering their modulation by ROS, holds promise for addressing infections, autoimmune conditions, and other immune-related disorders. In summary, while specific references for the direct influence of ROS on MAIT cell differentiation are not provided due to the evolving nature of research, the existing literature supports the idea that ROS likely contribute to the regulation and function of MAIT cells in the gastrointestinal tract (191). Additional research is necessary to clarify the intricate mechanisms and potential therapeutic applications. Subsequent investigations will unveil the dynamics of vitamin B2-derived metabolites *in vivo*, both under steady-state conditions and throughout the progression of infections and wound healing processes. Enhanced comprehension of MAIT cell triggers, coupled with the creation of stable agonists, will open avenues for harnessing the clinical potential of these plentiful T-cells.

## 9 NO and T-cell regulation

**Figure 9:**
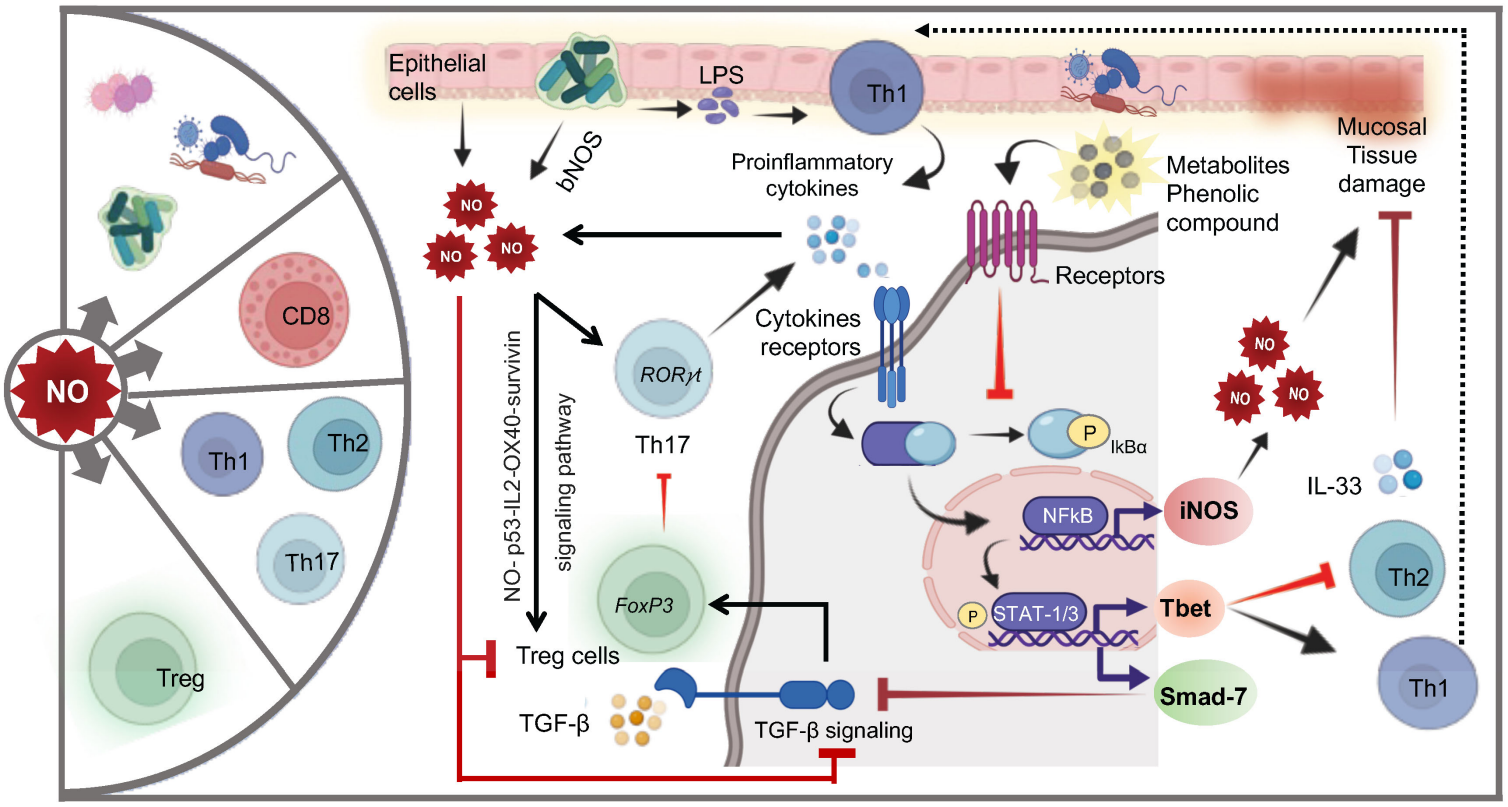
Cellular and molecular mechanisms of NO-mediated regulation of T cells. NO exerts profound effects on the composition of the gut microbiota and significantly influences various T-cell subsets. Epithelial cells and gut microbiota-produced NO promote the differentiation of inflammatory Th17 cells. Upon exposure to LPS, proinflammatory cytokines (PC: including IL-6, TNF-α, IFN-*γ*, IL-12, IL-17, and IL-18) secreted by Th1 or Th17 cells bind to their respective receptors, initiating NFkB activation and STAT1/3 phosphorylation, leading to NO production and regulation of Th1 and Th2 cells. High levels of NO can cause mucosal tissue damage, which may be repaired by IL-33-supporting Th2 cells. Phenolic metabolites reduce NO levels by inhibiting NFkB activation. NO also directly supports the expansion of regulatory T cells (Tregs) via the NO-p53-OX40 survivin signaling pathway. Additionally, upregulation of Smad-7 induced by STAT1 phosphorylation inhibits critical TGF-β signaling, a crucial anti-inflammatory pathway.

The dysfunction of the mucosal immune response is implicated in the pathogenesis of chronic inflammation of the gastrointestinal tract such as IBD, Crohn’s disease (CD), and ulcerative colitis (UC) (198). NO plays a multifaceted and crucial role in the intestine influencing smooth muscle relaxation, blood flow regulation, maintenance of epithelial barrier, microbial defense, and importantly immune regulation (15, 199). NO can be produced in eukaryotic cells by oxidation of L-arginine by NO synthase (15). Moreover, studies provide strong evidence suggesting that colonic epithelial cells serve as the primary source of NO production and NOS activity (**Figure 9**). This activity has been reported to be elevated in the mucosa of patients with UC (200). iNOS is essential to maintain a protective Th1/Th2 type of immune response via balanced production of proinflammatory and anti-inflammatory cytokine. However, dysregulated balance of immune response is often associated with inflammatory events.

Earlier research has indicated that the disturbance in the balance of cytokines is implicated in the initiation and advancement of IBD, CD and UC (198). iNOS is essential to maintain a balanced and protective Th1 and Th2 type of immune response and production of related cytokines (201). The elevated Th1 type of immune response is involved in the pathological process of CD (202). Accumulating evidence indicates increased production of NO following exposure to a pro-inflammatory stimulus like IFN-*γ*, TNF-α, IL-1β, IL-6 and microbial products like lipopolysaccharide (LPS) (**Figure 9**). The NF-κB a key transcription factor that exerts a crucial influence in regulating the survival, activation and differentiation of innate immune cells and inflammatory T- cells. The activation of NF-κB is a complex process that includes its translocation into the nucleus, where it can regulate the expression of various genes involved in proinflammatory process, including iNOS. The translocation of NF-κB is enabled by the phosphorylation of its inhibitor, IκBα. The degradation of IκBα is initiated through its site-specific phosphorylation by the IκB kinase (IKK) complex (203, 204). iNOS is highly expressed upon activation of the transcription factor NF-κB in response to pro-inflammatory cytokine. Interestingly, studies also suggests that sulfate and glucuronide metabolites in the intestine and phenolic compounds such as hydroxytyrosol and Tryrosol can also inhibits NF-ĸB-induced iNOS gene expression by activating Nrf-2 signaling (205, 206). These metabolites and their parent compound prevent the phosphorylation of IκBα, thereby preventing the activation of NF-ĸB (207). Moreover, the intestinal concentrate of these phenolic compounds also acts as antioxidant and anti-inflammatory agent contributing to its protective role (207, 208). During mucosal inflammation, the local immune response is primarily skewed toward a Th1 type, characterized by the release of cytokines TNF-α, IL-12, and IL-18 (209–212). Additionally, experimental murine colitis models demonstrated that neutralizing antibodies against TNF-α, IL-12, or IL-18 prevent the onset of colitis which further indicate that proinflammatory immune responses predominate in inflamed mucosa (210, 213). Transcription factor T-box expressed in T cells (Tbet), a key regulator of Th1 development via induction of IFN-*γ* and repression of Th2 cytokine. The upregulation of Tbet correlates with the production of IFN-*γ*, leading to the subsequent release of IL-12 and inducing Th1-mediated immunopathology in CD (212). In line with this, abnormalities in the levels of IFN-*γ* and IL-12 indicate a dysregulation of intestinal immunity. Higher levels of IFN-*γ* and IL-12 strongly correlate with high levels of NO in sera and colonic mucosa (214). IBD involve complex interactions between various cytokines and signaling pathways, contributing to chronic intestinal inflammation. Pro-inflammatory cytokines such as TNF-α, IL-1β, and IFN-γ activate NF-κB and STAT-1 and 3 pathways, leading to the upregulation of Smad-7 expression (215, 216). Elevated Smad-7 levels inhibit TGF-β signaling, a critical anti-inflammatory pathway, observed in IBD (217). Additionally, TGF-β production effectively suppresses experimental granulomatous colitis, highlighting its protective role (218). However, TGF-β knockout mice display increased NO production and iNOS expression, suggesting its regulatory function in dampening pro-inflammatory responses, including iNOS expression (**Figure 9**).

Conversely, an exaggerated Th2 immune response, characterized by anti-inflammatory cytokines dominance, may contribute to intestinal inflammation. Breaches in intestinal integrity trigger Th2 response, releasing IL-33, an alarmin that binds to ST-2 receptors on various immune cells. While IL-33 initially promotes wound healing, dysregulated IL-33/ST2 signaling is implicated in chronic intestinal inflammation (219, 220). Elevated IL-33 levels in IBD patients correlate with disease severity, and aberrant f-IL-33 isoform localization in intestinal epithelial cells further exacerbates inflammation. a substantial elevation of ST2 expression was also reported in the intestinal mucosa and sera from IBD patients (220). Furthermore, NO, a key mediator in mucosal damage and chronic inflammation, is regulated by cysteine oxidation. Cystine, a glutathione substrate, mitigates inflammation by preserving intestinal barrier integrity under oxidative stress. Overall, dysregulated cytokine signaling, particularly involving TGF-β and IL-33, along with NO-mediated oxidative stress, contribute significantly to the pathogenesis of IBD, highlighting potential therapeutic targets for intervention (221, 222) (**Figure 9**).

Th17 cells, characterized by the ROR*γ*t transcription factor and IL-17 cytokines, play a significant role in inflammatory diseases by contributing to NO production. Elevated levels of IL-17A in the inflamed mucosa and sera of IBD patients correlate with Th17-associated cytokines like IL-6 and IL-23, as well as with increased NO production (223, 224). Enhanced phosphorylation of STAT3 (p-STAT3) in the colonic mucosa triggers ROR*γ*t expression, promoting Th17 cell development (224). The upregulation of nitric oxide synthase 2 (NOS2) and p-STAT3 levels in the gut mucosa correlates with the severity of inflammation in IBD, highlighting their role in disease progression. While NO has both cytotoxic and immunoregulatory functions, its overproduction, often supported by IFN-*γ* and IL-17, exacerbates chronic inflammation in IBD. Elevated NO levels in the gut mucosa stimulate the production of pro-inflammatory cytokines like IL-17 by Th17 cells, forming a feedback loop that perpetuates inflammation. This bidirectional interaction between NO and Th17 cells contributes to the pathogenesis and progression of inflammatory disorders in the gut.

Treg cells are predominantly found in the thymus, but evidence suggests the intestine is vital for inducible Treg (iTreg) cell differentiation (225, 226). Treg cells are present throughout the body, including peripheral tissues like the intestine. The majority of Treg cells are indeed generated in the thymus during T-cell development, however, the intestine plays a crucial role in the differentiation and maintenance of iTreg cells, which are generated locally in response to antigens encountered in the gut environment. The gut microenvironment, with its diverse array of antigens, commensal bacteria, and metabolites, provides stimuli that promote the differentiation of naïve T-cell into iTreg cells. Metabolites derived from dietary components and microbial fermentation, as well as signaling pathways like the aryl hydrocarbon receptor (AHR) pathway, contribute to the induction of iTreg cells in the intestine (141, 148, 149). TGF-β, abundant in gut epithelium, promotes Foxp3^+^ Treg development and identified as a master regulator of intestinal microbiota and immune cell interactions (227, 228) (**Figure 9**). Imbalance between Tregs and Th1/Th17 cells correlates with severe intestinal inflammation (229, 230). NO, in conjunction with TCR activation, promotes the development and prolonged survival of Foxp3^+^ CD4^+^CD25^+^ Tregs from CD4^+^CD25^−^ T cells via p53, IL-2, and OX40 surviving signaling pathway (231). Moreover, research indicates that NO plays a pivotal role in FoxP3 regulation. Foxp3 is required for the adequate development and function of Tregs. Interestingly, experimental studies have shown that Foxp3 deficiency leads to a lack of CD4^+^CD25^hi^ Tregs, resulting in an inability to control the expansion of effector T-cells (232, 233). NO reduces FoxP3 expression and promotes Th1 cell differentiation by modulating TGF-β signaling (234). Studies show NO diminishes Foxp3 expression in MBP-primed T cells, while inhibiting iNOS or scavenging NO enhances Foxp3 expression (235). Both IL-6 and NO modulate TGF-β signaling, impacting T-cell differentiation toward Th17 or Th1 (235). Conversely, retinoic acid in the gut suppresses NO and IL-6, favoring Foxp3^+^ Treg over Th1/Th17 cells (234). This suggests a complex interplay between NO, TGF-β, and retinoic acid in regulating T-cell differentiation. Diet-derived metabolites like retinoic acid maintains gut barrier integrity and facilitates T cell homing into the gut (236, 237). Additionally, it enhances TGF-β-mediated induction of Foxp3^+^ Tregs by inhibiting Th17 development (238). Thus, NO exhibits dual roles, acting both as a cytotoxic agent and as an immunoregulator. Elevated NO levels are associated with chronic gut inflammation, driven by increased expression of pro-inflammatory cytokines like IFN-*γ* and IL-17, along with reduced levels of the anti-inflammatory cytokine TGF-β. These findings underscore the significant influence of NO on gut microbiota composition function, and T-cell regulation highlighting its potential role in shaping gut health and disease outcomes.

## 10 Future research directions

Future research in the field of gut redox and T-cell regulation holds significant promise for advancing our understanding of inflammatory and metabolic disorders and developing precision medicine approaches for their management. One key area for investigation is the elucidation of the complex interplay between gut redox balance, microbiota composition, and T-cell function. Understanding how alterations in gut redox homeostasis influence the composition and function of the microbiota, and in turn, impact T-cell responses, could provide insights into the pathogenesis of inflammatory and metabolic disorders. Moreover, identifying specific molecular pathways and signaling networks involved in mediating these interactions could reveal novel therapeutic targets for intervention. Challenges in this area include the need for comprehensive multi-omics approaches to capture the complexity of gut microbial communities and T-cell populations, as well as the integration of large-scale omics data with clinical phenotypes to identify predictive biomarkers of disease progression and treatment response. Cutting-edge technologies such as single-cell genomics and proteomics, coupled with advanced bioinformatic analyses, offer unprecedented opportunities to dissect the key molecular pathways underlying T-cell regulation in gut-related disorders. By leveraging these advanced tools, researchers can uncover novel molecular targets for precision medicine interventions aimed at modulating T-cell responses and restoring gut homeostasis in patients with inflammatory and metabolic disorders.

## Glossary

AHR: Aryl Hydrocarbon Receptor
AMPK: AMP-activated protein kinase
AP-1: Activator proten-1
APCs: Antigen-presenting cells
ARE: Antioxidant response element
ATP: Adenosine triphosphate
BHLHE40: Basic Helix-Loop-Helix Family Member E40
Blimp-1: B lymphocyte-induced maturation protein-1
Eomes: Eomesodermin
ER: Endoplasmic Reticulum
ETC: Electron transport chain
FAS: Fas Cell Surface Death Receptor
FPR1/2: Formyl peptide receptors 1 and 2
CAT: Catalase
CD: Crohn’s disease
CTLs: Cytotoxic T-lymphocytes
DCs: Dendritic cells
ER: Endoplasmic reticulum
ERK: Extracellular signal regulated kinase
ETC: Electron transport chain
FAO: Fatty acid oxidation
FcγR I/FcγR III: Fc receptors for IgG I/III
FFAR: Free fatty acid receptor
GI: Gastrointestinal
GLLC: Glutamate-cysteine ligase catalytic
GM: Gut Microbiota
GPR: G-protein receptors
GPR109A: G-protein-coupled receptor
GPx: Glutathione peroxidase
GR: Glutathione reductase
GSH: Glutathione
GST: Glutathione- S-S-Transferase
H_2_O_2_: Hydrogen peroxide
H3K9ac: Histone H3 acetylated in lysine 9
H_2_S: Hydrogen sulfide
HDAC: Histone deacetylase
HIF-1α: Hypoxia includible factor-1α
IBD: Inflammatory bowel disease
IL-2: Interleukin-2
ILCs: Innate lymphoid cells
iNKT: Invariant natural killer cells
JNK: c-Jun N-terminal kinase
Keap1: Kelch-like ECH-associated protein 1
LCA: Lithocholic acid
MAIT: Mucosal associated invariant T-cells
MAPK: Mitogen activated protein kinase
MEK: Mitogen activated kinase
MF: Myofibroblasts
MS: Multiple sclerosis
mTOR: mammalian Target of Rapamycin
NAC: N-acetyl cysteine
NF-κB: Nuclear factor-kappa B
NFAT: Nuclear factor of activated T-cells
NK: Natural killer cells
NO: Nitric oxide
NOS: N_2_O: Nitrous oxide
NOS: Nitric oxide synthase
NOX: NADPH oxidases
NOX-2: NADPH oxidase 2
NRF-2: Nuclear factor erythroid-2-related factor 2
Nur77: Nuclear receptor subfamily 4 group A member 1
OXPHOS: Oxidative phosphorylation
PD-L1: Programmed death ligand 1
PI3K: Phosphoinositide 3-kinases
PKC: Protein kinase C
PPP: Pentose phosphate pathway
RA: Rheumatoid arthritis
RAS: Rat sarcoma virus protein
RAF: Rapidly accelerated fibrosarcoma protein
ROS: Reactive oxygen species
RNS: Reactive nitrogen species
Runx3: RUNX Family Transcription Factor 3
SCAFs: Short-chain fatty acids
SFB: Segmented filamentous bacteria
SMC: Smooth muscle cells
SOD: Superoxide dismutase
Sp1/Sp3: Specificity protein 1 and 3
TAK1: TGFβ-activated kinase 1
TCM: Central memory T-cells
TCR: T-cell receptor
TEM: Effector memory T-cells
Tfh: T-follicular helper cells
TRM: Tissue resident memory T-cells
Tregs: Regulatory T-cells
TRX: Thioredoxin
Trx/TrxR: Thioredoxin/thioredoxin reductase
UC: Ulcerative colitis
